# DIX Domain Polymerization Drives Assembly of Plant Cell Polarity Complexes

**DOI:** 10.1016/j.cell.2020.01.011

**Published:** 2020-02-06

**Authors:** Maritza van Dop, Marc Fiedler, Sumanth Mutte, Jeroen de Keijzer, Lisa Olijslager, Catherine Albrecht, Che-Yang Liao, Marcel E. Janson, Mariann Bienz, Dolf Weijers

**Affiliations:** 1 Laboratory of Biochemistry, Wageningen University, Stippeneng 4, Wageningen, the Netherlands; 2 MRC Laboratory of Molecular Biology, Francis Crick Avenue, Cambridge CB2 0QH, UK; 3 Laboratory of Cell Biology, Wageningen University, Droevendaalsesteeg 1, Wageningen, the Netherlands

**Keywords:** cell polarity, plant development, DIX domain, dishevelled, SOSEKI, signalosome, protein oligomerization

## Abstract

Cell polarity is fundamental for tissue morphogenesis in multicellular organisms. Plants and animals evolved multicellularity independently, and it is unknown whether their polarity systems are derived from a single-celled ancestor. Planar polarity in animals is conferred by Wnt signaling, an ancient signaling pathway transduced by Dishevelled, which assembles signalosomes by dynamic head-to-tail DIX domain polymerization. In contrast, polarity-determining pathways in plants are elusive. We recently discovered *Arabidopsis* SOSEKI proteins, which exhibit polar localization throughout development. Here, we identify SOSEKI as ancient polar proteins across land plants. Concentration-dependent polymerization via a bona fide DIX domain allows these to recruit ANGUSTIFOLIA to polar sites, similar to the polymerization-dependent recruitment of signaling effectors by Dishevelled. Cross-kingdom domain swaps reveal functional equivalence of animal and plant DIX domains. We trace DIX domains to unicellular eukaryotes and thus show that DIX-dependent polymerization is an ancient mechanism conserved between kingdoms and central to polarity proteins.

## Highlights

• SOSEKI proteins are deeply conserved polar proteins in land plants
• A DIX domain mediates polymerization and polarization of SOSEKI proteins
• SOSEKI polymerization allows polar recruitment of an effector protein
• DIX-dependent polymerization is shared between animal and plant polarity proteins

## Graphical Abstract

**Figure:**
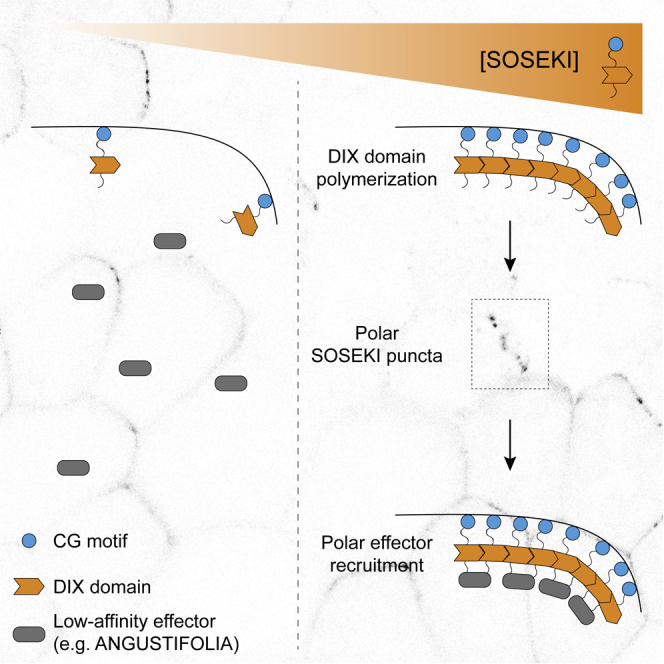

## Teaser

The identities of cell polarity determinants are not conserved between animals and plants; however, characterization of a DIX-domain containing protein in land plants reveals that the physical principles of polar complex assembly are preserved across eukaryotes.

## Introduction

Cell polarity is fundamental for the development of organisms across kingdoms of life. Polarity establishment involves the translation of directional cues into subcellular responses, such as local assembly of protein complexes or formation of outgrowths (St Johnston and Ahringer, 2010). In multicellular organisms, individual cell polarity additionally needs to integrate global organismal axes, and be coordinated among neighboring cells within a plane (Butler and Wallingford, 2017). Planar polarity ensures correct morphogenesis of tissues by regulating cell growth and differentiation, as well as through orienting structures protruding from single cells within epithelia, such as hairs and cilia (Butler and Wallingford, 2017). Despite the fundamental importance of cellular and planar polarity for multicellular life, it remains unclear how polarity-determining systems evolved in plants and animals. Do both kingdoms use similar mechanisms derived from their last single-celled common ancestor, or did each lineage evolve independent solutions to the same problem?

Various mechanisms conferring cell polarization have been uncovered in animals. A central component in planar cell polarity (PCP) depends on Wnt signaling, transduced by Dishevelled (DVL) (Strutt, 2002). Wnt ligands bind to Frizzled and one of several co-receptors, which leads to the activation of distinct branches of intracellular Wnt signaling, depending on the co-receptor (Angers and Moon, 2009). The best-studied branch is mediated by β-catenin which is stabilized upon Wnt binding to Frizzled and LRP5/6 co-receptor, and thus translocates to the nucleus to co-activate Wnt-dependent gene transcription (Gammons and Bienz, 2018). Non-canonical Wnt signaling does not involve β-catenin, but instead controls various cellular processes through other signaling intermediates (Strutt, 2002). Central to both canonical and non-canonical signaling is the Dishevelled hub protein, which assembles Wnt signalosomes by reversible head-to-tail polymerization of its DIX domain (Schwarz-Romond et al., 2007a). DIX filaments are cross-linked by dimerization of the Dishevelled DEP domain, generating three-dimensional dynamic structures (Gammons et al., 2016a), akin to phase-separated protein condensates (Schaefer and Peifer, 2019). Dishevelled thus attains a high local concentration that enables it to interact efficiently with low-affinity signaling effectors whose normal cellular concentration is too low to interact with unpolymerized Dishevelled (Bienz, 2014). Signaling effectors include Axin for canonical Wnt signaling and various other proteins in non-canonical signaling (Strutt, 2002, Yang and Mlodzik, 2015). While the importance of DIX domain-mediated polymerization is well-established in canonical Wnt signaling, its role in cell polarity signaling has not been tested. However, all available evidence suggests that the DIX domain may also be important for non-canonical signaling: for example, polar membrane-associated Dishevelled puncta (condensates) have been reported in *Drosophila* wing discs during PCP signaling (Axelrod, 2001, Strutt et al., 2016), in *C. elegans* B cells (Wu and Herman, 2007), and *Xenopus* embryos (Yamanaka and Nishida, 2007), and the DIX domain is required for polar localization in the latter two cellular contexts. Furthermore, upon deletion of its DIX domain, *Drosophila* Dsh behaves as a dominant-negative, producing planar polarity phenotypes in wings (Axelrod et al., 1998), indicating a function of this domain for PCP signaling.

Plants evolved multicellularity independently from animals, and may therefore use different polarity systems. Indeed, orthologs of the well-known polarity regulators from animals or yeast are thought to be missing from plant genomes (Kania et al., 2014), with the exception of the Rho-of-Plants (Rop) proteins (Yang, 2008) that are important for cell morphogenesis (Yang and Lavagi, 2012). However, a role for Rop proteins in polarization of dividing cells has not yet been found. Several plant-specific proteins have been linked to polarity because of their accumulation at one side of the cell. For example, PIN auxin hormone transport facilitators (Gälweiler et al., 1998, Kania et al., 2014), Boron transporters NIP5;1 and BOR1 (Takano et al., 2010), POLAR scaffold protein (Pillitteri et al., 2011), SGN1 protein kinase (Alassimone et al., 2016), and CASP scaffold proteins (Roppolo et al., 2011) all localize to specific sides of plant cells. However, their localization is readily perturbed by experimental manipulations of transport systems or cellular trafficking (Kania et al., 2014) and often depends on tissue context and developmental stage. As such, most currently known polar proteins are likely clients or readouts of polarity systems, rather than integral components of polarity-generating pathways. Some polar proteins, such as the BASL scaffold protein (Dong et al., 2009) and its partner protein BRXL2 (Rowe et al., 2019), have been shown to regulate cell polarity or asymmetric cell division. However, BASL is expressed in specific tissues and cell types exclusively of flowering plants, which makes it unlikely that it is a constituent of a universal polarity-generating mechanism. Such a mechanism may be expected to be conserved in early-diverging land plants such as mosses or liverworts; however, little is known about cell and tissue polarity in these organisms. In fact, the only polar protein that has been found in these species is the PINA protein of the moss *Physcomitrella patens* that shows polar localization in tip-growing cells, and bi-polar localization in leafy tissues (Viaene et al., 2014) distinct from the unique polar patterns in flowering plants (Gälweiler et al., 1998, Kania et al., 2014). In summary, the mechanisms that establish and integrate polarity in plants remain elusive, and it is even less clear whether plant polarity systems bear any similarity to polarity-generating signaling pathways in animals.

We recently discovered a family of five paralogs called SOSEKI (SOK1–SOK5) in the flowering plant *Arabidopsis thaliana*. Each of these proteins displays robust polar edge localization in multiple cell types throughout development. Polar localization of SOSEKI proteins involves two conserved domains: a central domain required for membrane association that dictates localization to polar edges, and an N-terminal domain required for focused localization at these edges (Yoshida et al., 2019). The N-terminal domain was proposed to contain a DIX domain-like fold, hinting at similarities between animal and plant cell polarity systems. Here, we discover orthologs of SOSEKI proteins across all land plants, and we show that paralogs in two bryophyte species also display polar edge localization. Furthermore, we use structural and biochemical analysis to establish that this ancient protein family contains a bona fide DIX domain—constituting the closest structural relatives of the Dishevelled DIX domain—and that these domains, like Dishevelled DIX, undergo concentration-dependent head-to-tail polymerization. We also use cross-kingdom assays to reveal functional equivalence between Dishevelled and plant DIX domains. Notably, the likely origin of DIX domains can be traced to unicellular eukaryotic ancestors. Collectively, our insights suggest an ancient origin of a phase-separating biochemical paradigm underlying cell polarity in animals and plants.

## Results

### SOSEKI Proteins Are Shared across Land Plants

The genome of the flowering plant *Arabidopsis thaliana* encodes five SOSEKI proteins, each of which shows polar localization during development (Yoshida et al., 2019). To identity other SOSEKI proteins in the plant kingdom, we searched the OneKP dataset (Matasci et al., 2014, Wickett et al., 2014) using a bioinformatic pipeline as previously described (Mutte et al., 2018). This dataset encompasses RNA sequencing (RNA-seq) transcriptome assemblies from more than a thousand plants species, including both land plants and their aquatic sister group, the green algae (Matasci et al., 2014, Wickett et al., 2014). Each of the five *Arabidopsis SOSEKI* paralogs (AtSOK1–AtSOK5) was used as query for BLAST searches of the OneKP dataset. To recover more distantly related sequences, we also searched the genome of the early-diverging liverwort plant *Marchantia polymorpha* (Bowman et al., 2017). This identified a single *SOSEKI-*like sequence (*MpSOK*), which was used for additional OneKP searches.

**Figure 1:**
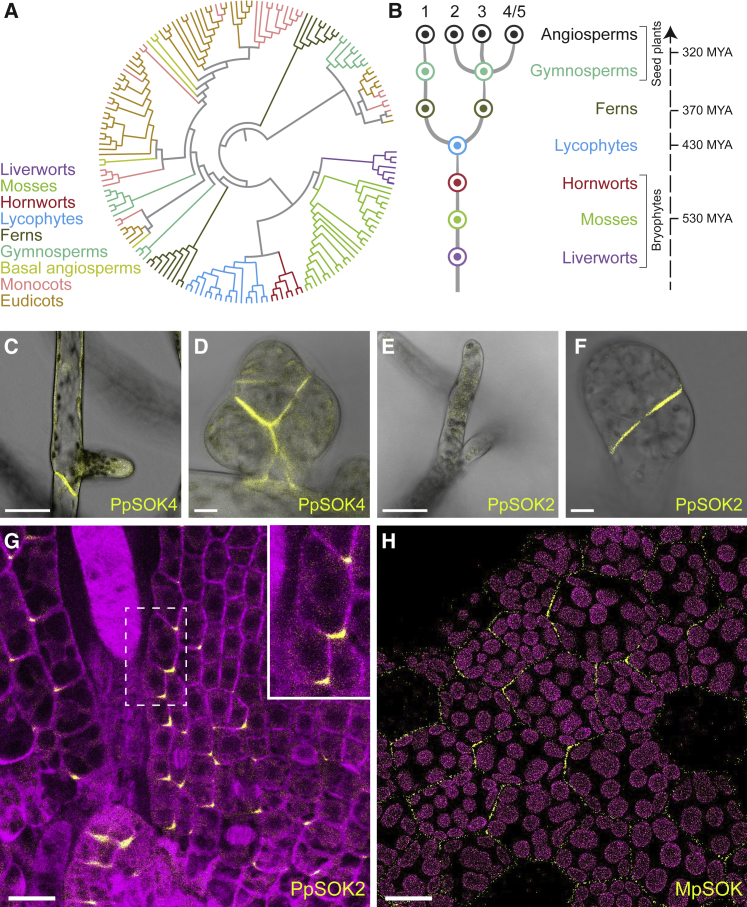
SOSEKI Proteins Are Ancient Polarity Proteins (A) Maximum Likelihood phylogenetic tree of SOSEKI sequences in land plants. (B) Inferred scenario of *SOSEKI* gene family evolution based on extended phylogeny. *Dots*, inferred ancestral gene number at each point of divergence of major land plant groups; numbers on top, *Arabidopsis SOSEKI* gene nomenclature; numbers on the right, estimated time of divergence in millions of years (MYA). (C–G) Representative confocal images of *Physcomitrella patens* expressing (C and D) PpSOK4-Citrine in (C) protonema or (D) gametophore bud, or (E–G) PpSOK2-Citrine in (E) protonema, (F) gametophore bud, or (G) leafy gametophore (magenta, mCherry-Tubulin). (H) Localization of Citrine-MpSOK, (yellow) expressed from the *35S* promoter in a *Marchantia polymorpha* gemma (magenta: chloroplast autofluorescence). Scale bars, 25 μm (C–G), 10 μm (H). See also Figure S1 and Table S1.

*SOSEKI*-related sequences are widespread throughout land plants, and we thus subjected them to phylogenetic analysis. The rich species sampling in the OneKP dataset, with multiple species in each major taxonomic clade, allowed us previously to infer the plausible number of ancestral gene copies within a number of gene families at the divergence of each lineage (Mutte et al., 2018). Applying this strategy to SOSEKI proteins, we found these to be limited to land plants, and no clear homologs could be identified in algal sister groups (Figures 1A and 1B). Thus, it is likely that SOSEKI proteins are limited to land plants, but more genome-based information will be required to confirm their absence in algae. Indeed, a single *SOSEKI* ancestor must have existed until a first duplication gave rise to *SOK1* and *SOK2*–*SOK5* precursors (*Arabidopsis* nomenclature) in the common ancestor of ferns and seed or flowering plants (Figures 1A and 1B). Subsequent duplications in flowering plants increased the number of paralogs (Figure 1A and 1B). Because RNA-seq transcriptome assemblies tend to miss genes that are weakly expressed in sampled tissue, we also searched curated genome sequences of 107 angiosperms, seven gymnosperms, a single lycophyte, and two bryophyte species (https://bioinformatics.psb.ugent.be/plaza/). Strikingly, none of the investigated land plant species lacks *SOSEKI* genes (Table S1), which indicates a fundamental function of these genes in all land plants.

### SOSEKI Proteins Are Ancient Polar Proteins

**Figure S1:**
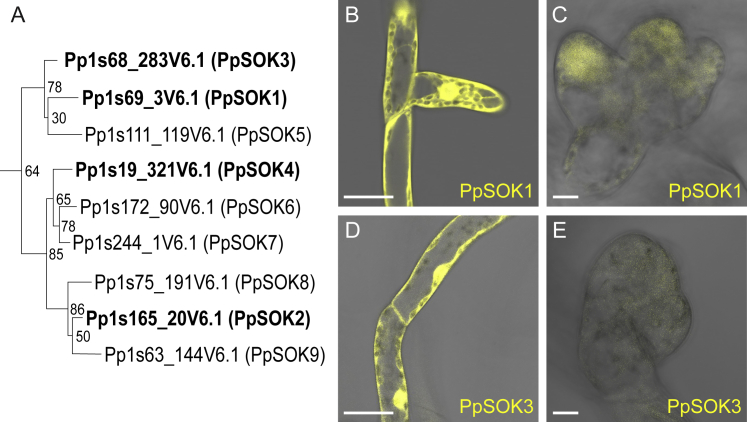
Phylogeny and Localization of PpSOK Proteins, Related to Figure 1 (A) Maximum Likelihood phylogenetic tree of SOSEKI sequences in *Physcomitrella patens*. Proteins are indicated as unique gene identifiers using nomenclature from version 3.3 of the *Physcomitrella* genome assembly, as well as a PpSOK number. Nodes are marked with bootstrap support values across 1000 replicates. (B–E) Representative confocal images as in main Figures 1C–1G, revealing subcellular localizations of PpSOK1-Citrine (B and C) and PpSOK3-Citrine (D and E) in protonema (B and D) and gametophore buds (C and E), as indicated in panels. Scale bars, 25 μm in (B–E).

All five *Arabidopsis* SOSEKI proteins show polar localization, but it is unclear whether this reflects an ancestral or derived property. To address this, we determined the subcellular localizations of SOSEKI proteins in two bryophyte species. We first localized four of the nine SOSEKI proteins found in the moss *Physcomitrella patens* (named *PpSOK*). All *PpSOK* genes, including one encoding a truncated protein, are derived from a single ancestral bryophyte gene by duplications within the moss ancestor, and are thus all co-orthologous to *AtSOK* genes (Figure S1A). We selected four genes for which expression had been documented (Ortiz-Ramírez et al., 2016) (http://bar.utoronto.ca/efp_physcomitrella/cgi-bin/efpWeb.cgi) for genomic tagging with a C-terminal mCitrine fluorescent protein by homologous recombination.

Three of the four PpSOK proteins are expressed during the filamentous stage (protonema) of *Physcomitrella* development. In the tip-growing and branching cells, two of these (PpSOK1, PpSOK3) are diffuse throughout the cytosol and nucleus (Figures S1B and S1D), while the truncated PpSOK4 marks the newly formed cell walls (Figure 1C). The fourth (PpSOK2) is only expressed after the switch from filamentous to three-dimensional growth (Figure 1E and 1F). In young gametophore buds, PpSOK2 shows coordinated localization limited to the lower inner edge of bud cells (Figure 1F). In the leafy gametophores that develop from these buds, coordinated polarized PpSOK2 localization points to the leaf base and away from the leaf margin (Figure 1G). Following the switch from filamentous to three-dimensional growth, we detect a weak cytosolic signal for PpSOK1 and PpSOK3 in young gametophore buds (Figures S1C and S1E) while PpSOK4 remains localized to the cell plate (Figure 1D). Because the apical cells in young buds divide in a polarized fashion (Harrison et al., 2009), localization of SOSEKI proteins to newly formed walls in these buds will result in basal polar localization in the apical-most cell (Figure 1D).

Two of the four PpSOK proteins did not show polar localization, which is likely a consequence of loss of polarity after gene duplications. However, it is also possible that the ancestral state was non-polar and some of the PpSOK proteins and all *Arabidopsis* SOK proteins independently gained polarity. To test if polarity is retained in a species that has only a single SOSEKI protein, we localized the single *Marchantia polymorpha* MpSOK protein. Because fluorescence was too weak to be detected when Citrine was fused to the C terminus of MpSOK in the context of a genomic fragment with ∼4 kb of endogenous promoter (not shown), we expressed a Citrine-MpSOK protein from the *35S* promoter. In these lines, we observed clear polar localization in gemmae (Figure 1H), that appeared coordinated among cells.

In summary, the only *Marchantia* SOSEKI protein, and two of the four tested *Physcomitrella* SOSEKI proteins show polarized localizations similar to their *Arabidopsis* counterparts. Notably, these three species are separated by 450 million years of evolution (Rensing et al., 2008), and therefore polar localization appears to be an ancestral property. Thus, SOSEKI proteins belong to an ancient polarity system common to all land plants.

### SOSEKI Polarity Relies on Highly Conserved Elements

**Figure 2:**
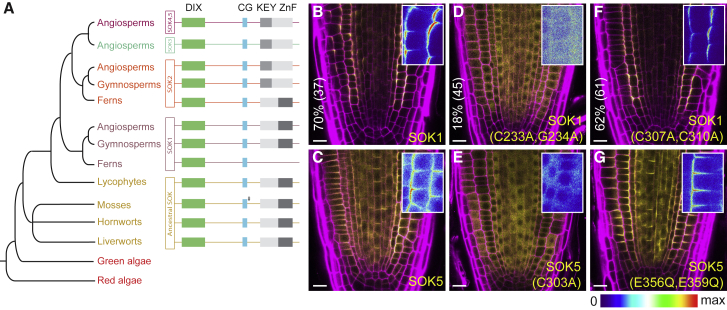
SOSEKI Proteins Have a Characteristic Domain Architecture (A) Phylogeny and conserved domain topology of land plant SOSEKI proteins; #, degenerated motif. (B–G) Representative confocal images of root tips, revealing subcellular localizations of WT (B), C233A/G234A mutant (D), C307A/C310A mutant (F), SOK1-YFP and wild-type (C), C303A mutant (E), and E356Q/E359Q mutant (G) SOK5-YFP as indicated in panels. Insets: magnifications in false color, highlighting apico-lateral SOK1-YFP and lateral SOK5-YFP localizations; underneath, false color scale; magenta, propidium iodide counterstain. The percentage of transgenic individuals with cell division orientation defects and number of transgenics (%/n) are indicated on the left of (B, D, and F). Scale bars, 10 μm (B–G). See also Figure S2.

**Figure S2:**
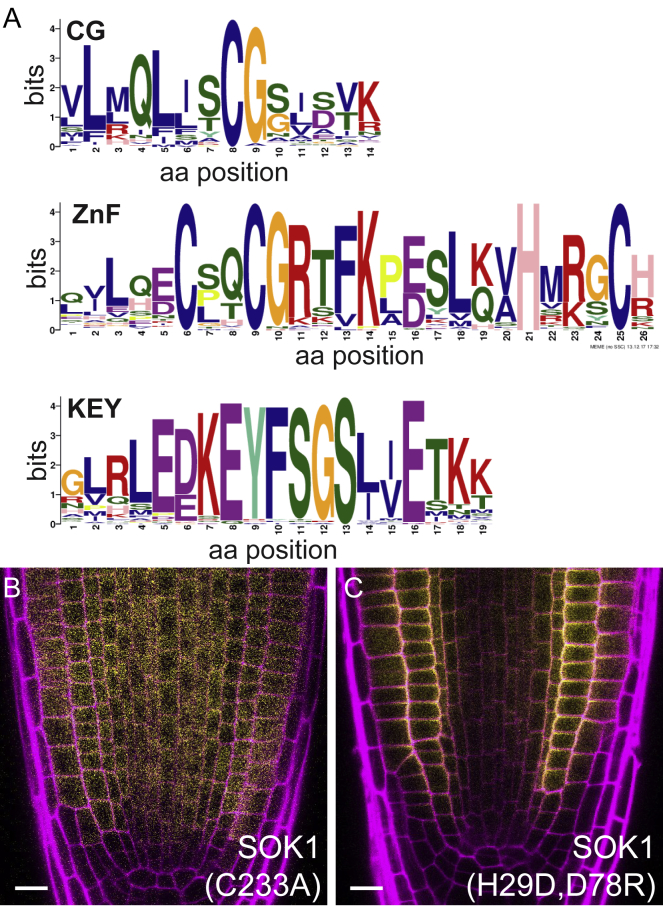
Conserved SOSEKI Elements and Localizations of Mutant SOK1-YFP, Related to Figures 2 and 3 (A) Amino acid logos of conserved elements in plant SOSEKI proteins. (B and C) Representative confocal images as in main Figures 2B–2G, revealing subcellular localizations of C233A (B) and H29D/D78R (C) mutant SOK1-YFP, as indicated in panels. Magenta: Propidium Iodide counterstain. Scale bars in (B and C), 10 μm.

Mining the full set of land plant SOSEKI sequences for conserved elements, we uncovered a common domain topology, including a prominent N-terminal signature domain (part of the DUF966 Interpro domain; https://www.ebi.ac.uk/interpro/entry/IPR010369) (Figure 2A). Further downstream, SOSEKI proteins exhibit an invariant CG motif (Figures 2A and S2A) predicted to be a target site for palmitoylation (Ren et al., 2008), thus potentially conferring membrane association. Within their C termini, they exhibit a conserved domain that can be divided into two distinct subclasses that are mutually exclusive among different orthologs and paralogs (Figures 2A and S2A). One of these subclasses bears a C2HC zinc finger (ZnF) signature, found in bryophyte and lycophyte SOSEKI and in the AtSOK1 clade of vascular plants, while the other subclass, found in AtSOK2–AtSOK5 orthologs of vascular plants, bears a central KEY motif (Figures 2A and S2A). However, several residues are highly conserved in both elements (e.g., two invariant charged residues and five hydrophobic residues, including one of the ZnF signature cysteines; Figure S2A), suggesting a shared structural fold (to be called KEY/ZnF domain). Notably, early land plants exclusively encode ZnF-bearing SOSEKI orthologs, suggesting that AtSOK1 typifies the ancestral protein.

We previously used a misexpression system based on the *RPS5A* promoter to demonstrate the functional relevance of the N-terminal DUF966 domain in AtSOK1 (Yoshida et al., 2019). When misexpressed, SOK1 localizes to polar edges in any cell it is expressed in, and this misexpression induces aberrations in cell division orientation (Figure 2B; 70% of independent transgenics show at least one abnormal cell division plane in median cross-section; n = 37), which serves as a readout for biological activity of the SOK1 protein. To examine the functions of SOSEKI elements, we misexpressed tagged AtSOK1 (SOK1-YFP) and AtSOK5 (SOK5-YFP) with the same system, which mediates ubiquitous polar localization across the root meristem (Figures 2B and 2C). Point mutations of the CG motif cause delocalization of SOK1-YFP (C233A and C233A/G234A) and SOK5-YFP (C303A) from cell membranes (Figures 2D, 2E, and S2B), indicating that this motif is essential for membrane association. Likewise, the C233A and C233A/G234A mutations in SOK1 also impaired its ability to alter cell division planes (Figure 2D; 18%; n = 45). However, we were unable to detect any changes in the localization or biological activity of SOK1-YFP bearing a mutant ZnF motif (C307A/C310A; Figure 2F; 62%; n = 61) nor in SOK5-YFP bearing a mutant KEY motif (E356Q/E359Q; Figure 2G), suggesting that these motifs are either dispensable for polar localization in our overexpression system, or that their function is redundant with another SOSEKI element. We conclude that the two most highly conserved elements of SOSEKI proteins, their DIX-like domain and their CG motif, have key functions in polar localization of SOSEKI proteins.

### SOSEKI DIX Is Biochemically and Structurally Equivalent to Dishevelled DIX

**Figure 3:**
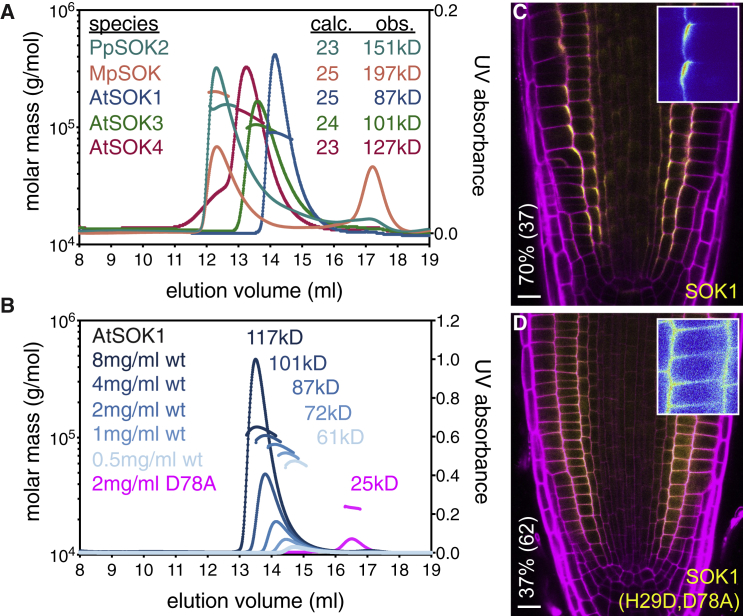
SOSEKI DIX Polymerization Depends on Protein Concentration (A and B) SEC-MALS of (A) purified DIX domains from *Arabidopsis* (AtSOK1, AtSOK3, AtSOK4), *Marchantia* (MpSOK), and *Physcomitrella* (PpSOK2), and (B) WT or D78A mutant AtSOK1 DIX at increasing protein concentrations as shown in graph. Curves, elution profiles; line traces, molar masses, as derived from MALS; calc, calculated molecular masses of monomers; obs, observed molecular masses of proteins. (C and D) Representative confocal images of WT (C) and polymerization-defective H29D/D78A mutant (D) SOK1-YFP, as in Figures 2B–2G. The percentage of transgenic individuals with cell division orientation defects and number of transgenics (%/n) are indicated on the left of (C) and (D). Scale bars, 10 μm (C and D). See also Figure S2.

Previously, we found that the N-terminal part of the DUF966 domain of AtSOK1 is capable of homo-dimerization, and that it may resemble the DIX domains of Dishevelled and Axin, based on secondary structure predictions (Yoshida et al., 2019). The Dishevelled DIX domain undergoes dynamic head-to-tail polymerization, which results in filamentous assemblies that can be observed by electron microscopy and in protein crystals (Madrzak et al., 2015, Schwarz-Romond et al., 2007a). Polymerization can also be detected by equilibrium ultracentrifugation or size exclusion chromatography coupled to multi-angle light scattering (SEC-MALS) (Fiedler et al., 2011, Liu et al., 2011, Schwarz-Romond et al., 2007a). To determine whether the previously observed homo-dimerization of the putative DIX domain of AtSOK1 reflects its ability to form polymers, we purified the domains from various *Arabidopsis* and *Physcomitrella* SOSEKI proteins, and from the single *Marchantia* SOSEKI paralog, for analysis by SEC-MALS. Indeed, each of these formed oligomers (Figure 3A), similarly to their animal counterparts (Fiedler et al., 2011, Liu et al., 2011, Schwarz-Romond et al., 2007a). Importantly, oligomerization is concentration-dependent, with higher concentrations leading to larger oligomers (Figure 3B).

Next, we attempted to crystallize these domains, but were unable to obtain diffracting crystals, presumably because of structural heterogeneity generated by rapid polymerization during crystallization. Note that no structure of a wild-type (WT) Dishevelled DIX has been determined as yet, although multiple structures of domains bearing polymerization-blocking point mutations have been solved (Liu et al., 2011, Madrzak et al., 2015, Yamanishi et al., 2019a, Yamanishi et al., 2019b). We thus used the structure of the human Dishevelled-2 (DVL2) DIX domain (Madrzak et al., 2015), to design a set of point mutations in the AtSOK4 domain that are predicted to attenuate its polymerization (see below). Crystallizing several of these mutations, we succeeded in obtaining diffracting crystals for D85A (equivalent to D78A in AtSOK1), which severely attenuates polymerization (Figure 3B). We thus solved the structure of this domain at 1.7 Å resolution (Table S2), following labeling with selenomethionine to determine phasing.

**Figure 4:**
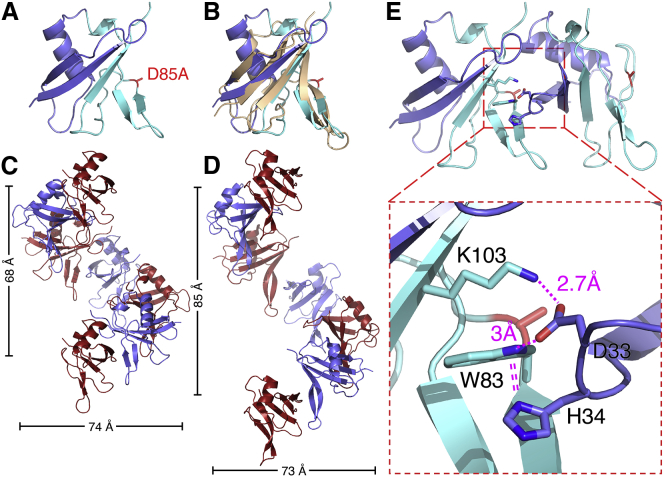
AtSOK4 Contains a Bona Fide DIX Domain (A) Ribbon diagram of SOK4 DIX (blue, head; cyan, tail); D85A, polymerization-disabling mutation facilitating crystallization (see text). (B) Overlay of AtSOK4 DIX (blue) and DVL2 DIX (4WIP; wheat). (C and D) Helical polymers of (C) AtSOK4 DIX and (D) DVL2 DIX as seen in crystals, with widths and lengths of helical turns indicated. (E) Interface between AtSOK4 DIX tail and head; dashed box: magnified view, with close hydrogen bond between D33 (head) and K103 and W83 (tail) and π-stacking between H34 (head) and W83 (tail) indicated (dotted lines). See also Figure S3 and Table S2.

**Figure S3:**
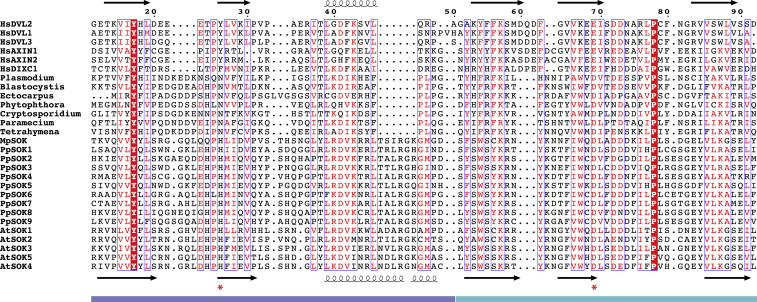
DIX Domain Conservation across Kingdoms, Related to Figure 4 Sequence alignments of the DIX proteins used in this study, with β strands (arrows) and α helices (spirals) indicated. Head and tail surfaces are marked by blue and cyan, respectively. The D and H residues that were mutated in SOK1 and SOK4 are marked by red asterisk.

Like DVL2 DIX, the AtSOK4 domain adopts a ubiquitin-like fold, with four β-strands and one α-helix (Figure 4A). Strikingly, its α-backbone almost perfectly superimposes on that of DVL2 DIX, with a root-mean-square deviation (RMSD) of 1.74 Å (Figure 4B). This was rather unexpected, given the limited primary sequence conservation between the two domains (19/93 residues; Figure S3). Indeed, this value is significantly lower than the RMSD between DVL2 DIX and the PB1 domain (e.g., of p62, 2.2 Å), the closest structural relative of DIX (Bienz, 2014), or between DVL2 DIX and the DIX-like domain of the neuro-pathogenic protein TDP-43 (2.26 Å), a recently discovered relative of DIX (Afroz et al., 2017). Like other DIX domains (Figure 4D), AtSOK4 DIX forms a helical filament in the crystal, via head-to-tail interactions of individual monomers (Figure 4C). The interface between individual monomers exhibits considerable hydrophobicity, and close hydrophobic interactions (e.g., W83-F81, W83-H34, W83-H36, Y84-F37) are likely to be crucial for the DIX-DIX interaction, although hydrogen bonds (e.g., D85-H36) are also likely to contribute (Figure 4E; recall that D85 was mutated for structure determination). Nearly all these residues are fully conserved, or even invariant, among land plant DIX domains (Figure S3), suggesting that they are critical in mediating polymerization. We conclude that the land plant SOSEKI proteins contain bona fide DIX domains that are structurally and biochemically equivalent to animal DIX domains.

### DIX-Dependent Polymerization Is Required for Polar Localization

To determine whether DIX-dependent SOSEKI polymerization is functionally relevant *in vivo*, we designed point mutations in the AtSOK1 DIX interface that are expected to impair polymerization. We confirmed this by SEC-MALS, whereby the most severe mutations produce monomers (Figure 3B). Using our root misexpression assay to test some of these polymerization-disabling mutations (H29D/D78A and H29D/D78R) for their effects on SOK1-YFP in transgenic *Arabidopsis* plants, we found that both double-mutations strongly reduced SOK1 polar localization (Figures 3C, 3D, and S2C). Likewise, polymerization-impairing mutations also reduced the ability of SOK1 protein to alter cell division planes in the root (Figure 3D; 37%; n = 62). Thus, the DIX-dependent polymerization of AtSOK1 is crucial for its polar localization and biological activity in developing *Arabidopsis* plants.

### Functional Equivalence between Dishevelled and Plant DIX Domains

**Figure 5:**
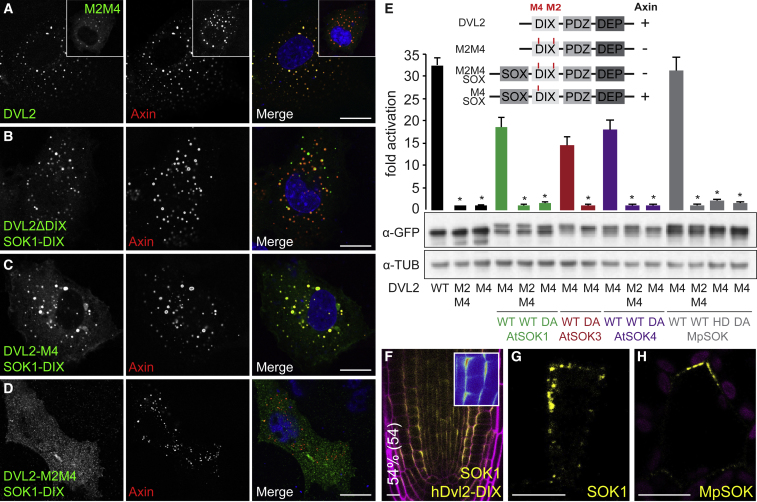
DVL2 and SOK DIX Domains Are Functionally Equivalent (A–D) Representative confocal images of COS-7 cells co-expressing FLAG-Axin with wild-type GFP-DVL2 (A), or various GFP-SOK1-DIX chimeras without DVL2 DIX domain (B) or with M4 (C) or M2/M4 (D) mutations in DVL2-DIX, as indicated in panels, fixed and stained with antibodies against FLAG (red in merges) or GFP (green in merges). Inset: DVL2M2M4-GFP co-expressed with FLAG-Axin. (E) SuperTOP reporter assays in Dishevelled null mutant HEK293T cells expressing DVL2-GFP or various GFP-SOK1-DIX chimeras, as indicated below graph (see also cartoon in Figure 5E, and main text; SOK DIX domains are abbreviated SOX here); shown are fold induction levels relative to DVL2-M2M4; data represent the mean ± SEM (n = 6); ^∗^p < 0.0001 (one-way ANOVA) for comparison to respective WT DIX domain. Underneath: corresponding western blots indicating expression levels (TUB, β-tubulin). (F) Representative confocal images of SOK1-YFP carrying the DVL2 DIX domain, as in Figures 2B–2G. The percentage of transgenic individuals with cell division orientation defects and number of transgenics (%/n) are indicated on the left. (G and H) High-resolution images of (G) *Arabidopsis* SOK1-YFP puncta in an early *Arabidopsis* embryo, expressed from endogenous promoter, and (H) *Marchantia* MpSOK in gemma. Scale bars, 5 μm (A–D), 10 μm (F), 5 μm (G), 10 μm (H).

We next explored the functional equivalence between Dishevelled and *Arabidopsis* DIX, using a previously described cell-based assay (Yamanishi et al., 2019b) allowing us to test whether *Arabidopsis* DIX could substitute for DVL2 DIX. In mammalian cells, DVL2 signalosomes are detectable as dynamic subcellular puncta (Figure 5A) whose formation is abolished by polymerization-disabling point mutations in the DIX domain (Bilic et al., 2007, Schwarz-Romond et al., 2005). These puncta confer Wnt/β-catenin signaling through the recruitment of the Axin effector (Schwarz-Romond et al., 2007b). Deletion of the DIX domain (DVL2ΔDIX) (Schwarz-Romond et al., 2005) or polymerization-disabling mutations (M2M4; Figure 5A) (Yamanishi et al., 2019b) abolish puncta formation. These puncta can, however, be restored by inserting *AtSOK* DIX into DVL2ΔDIX (SOK1 chimera called DVL2ΔDIX-SOK1-DIX; Figure 5B). Yet, these SOK1-DIX puncta do not colocalize with co-expressed Axin (Figure 5B) presumably because the Axin DIX domain cannot heteropolymerize with *AtSOK1* DIX owing to pronounced differences in their polymerization interfaces (Schwarz-Romond et al., 2007a) (Figure 5B). However, Axin colocalization can be restored by the addition of a point mutant DVL2 DIX domain that retains a normal tail surface (M4; Figure 5C) albeit not with a double mutant DVL2 DIX domain whose head and tail surfaces bear polymerization-disabling mutations (M2M4; Figure 5D).

As expected from its co-localization with Axin (Figure 5C), the M4-bearing SOK1 chimera restores β-catenin-dependent signaling activity in Dishevelled null mutant HEK293T cells (Gammons et al., 2016b), as can be monitored by a β-catenin-dependent transcriptional luciferase reporter called SuperTOP (Veeman et al., 2003), similarly to WT DVL2 (Figure 5E). However, this activity is essentially abolished if the chimera bears a double mutant M2M4 DVL2 DIX domain or a D78A mutant AtSOK1 DIX domain (Figure 5E). The same is true for chimeras bearing DIX domains from AtSOK3, AtSOK4, or MpSOK (*Marchantia polymorpha* SOSEKI) whose signaling activities are also inactivated by corresponding polymerization-disabling D > A mutations (Figure 5E). These cross-kingdom complementation assays confirm that a single intact DVL2 DIX surface is required for Axin interaction and β-catenin-dependent signaling, as previously shown (Yamanishi et al., 2019b). They establish the functional equivalence of SOSEKI and DVL2 DIX domains with regard to signalosome assembly and activity.

We next asked if, in a reciprocal experiment, the human DVL2 DIX domain would be able to replace the *Arabidopsis* SOK1 DIX domain. A chimeric SOK1 protein, in which its DIX domain was replaced by DVL2 DIX, was misexpressed in *Arabidopsis* as a C-terminal YFP fusion using the *RPS5A* promoter. The chimeric SOK1 protein localized to its polar edge (Figure 5F) in a manner that was indistinguishable from WT SOK1 (Figures 2B and 3C), and clearly distinct from the apolar SOK1 DIX mutant (Figure 3D). Thus, the biochemical properties of the polymerizing DIX domains in SOK1 and DVL2 are equivalent in the context of polarization (SOK1), puncta formation and canonical Wnt signaling (DVL2). Intriguingly, the human DVL2 DIX domain could also functionally replace the SOK1 DIX domain in altering division orientation (Figure 5F; 54%; n = 54), which suggests that this function requires DIX domain polymerization, but not interactions of the DIX domain with other plant-specific factors.

Dishevelled forms puncta, both in cultured cells (Figure 5A) and in imaginal disc cells where it acts in PCP signalling (Axelrod, 2001, Strutt et al., 2016). Given the equivalence of their DIX domains, we asked if SOK1 can also form puncta. SOK1 is first expressed in the early embryo (Möller et al., 2017, Yoshida et al., 2019), and embryonic cells thus allow visualizing early stages of polarization of SOK1. Indeed, high-resolution imaging of SOK1-YFP, expressed from its natural promoter, in young *Arabidopsis* embryos showed clear puncta at the membrane, and enriched in the polar edge domain (Figure 5G). We also examined *Marchantia* MpSOK localization at high resolution and found similar polar puncta (Figure 5H). Thus, the first step toward polar SOK localization appears to involve the formation of puncta, mediated by DIX domain polymerization, which likely subsequently coalesce.

### Polymerization-Dependent Recruitment of the ANGUSTIFOLIA Effector

We have shown that the DIX-dependent polymerization of SOSEKI proteins is crucial for their polar localization in plants and for signaling activity in functional cross-kingdom complementation assays in mammalian cells. In animal cells, polymerization of Dishevelled results in a drastic increase (>1,000-fold) in local concentration, which increases its avidity for Axin and thus enables recruitment of this low-affinity signaling effector (Bienz, 2014). Thus, DIX polymerization is required to overcome the weak affinity between the DIX domains of Dishevelled and Axin (K_d_ in the mid-micromolar range) (Fiedler et al., 2011; Yamanishi et al., 2019b).

**Figure S4:**
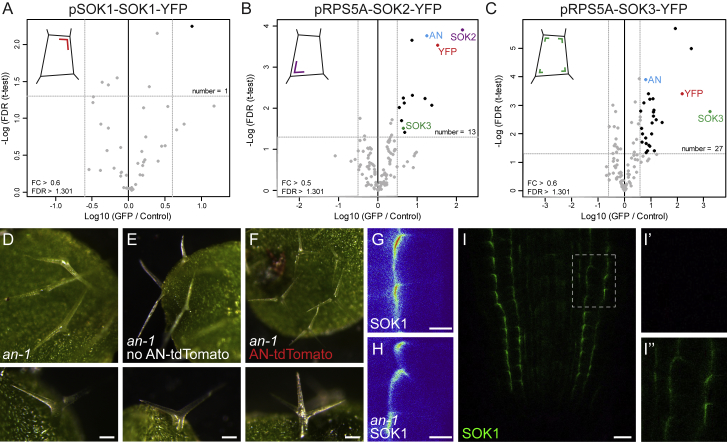
Identification of AtSOK-Interacting Proteins, Related to Figure 6 (A–C) Identification of proteins co-purifying with AtSOK1-YFP (A), RPS5A-AtSOK2-YFP (B), and RPS5A-AtSOK3-YFP (C), as in main Figure 6A; only one significant protein was identified in (A), using a threshold of > 1.301 (FDR) and > 0.3 FC. Note that AN is identified in SOK2 and SOK3 complexes, and SOK3 is co-purified with SOK2. (D–F) Complementation of the *an-1* mutant by AN-tdTomato; (D and E) two-branched trichomes in (D) *an-1* or (E) non-transgenic siblings segregating in a heterozygous AN-tdTomato line; (F) three-branched trichomes in a transgenic AN-tdTomato leaf. Lower panels in (D–F) show close-up of a single trichome. (G and H) Localization of RPS5A-SOK1-YFP in wild-type (G) and *an-1* (H) root tip. (I) Representative confocal image of root tip co-expressing SOK1-YFP (green) and AN-tdTomato; (I’) lack of YFP bleed-through signal in tdTomato channel; (I’’), YFP fluorescence. Scale bars, 100 μm in (D–F), 5 μm in (G and H) and 10 μm in (I).

**Figure 6:**
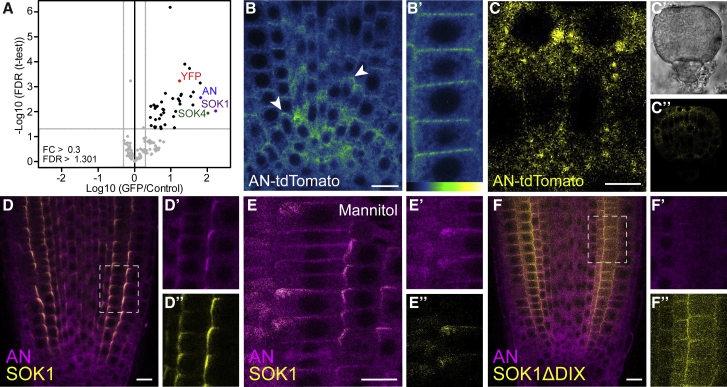
Polar Accumulation of AN Depends on the SOSEKI DIX Domain (A) Proteins co-purifying with SOK1-YFP stably expressed in transgenic RPS5A-SOK1-YFP *Arabidopsis* roots; x axis, protein enrichment (fold-change [FC]) of proteins co-purified with SOK1-YFP relative to samples from non-transgenic roots; y axis, statistical significance of enrichment (p value of false discovery rate [FDR]; Student’s t test; n = 3 replicates). 41 proteins were >0.3-fold FC and >1.301 FDR, including SOK1, SOK4, and AN. (B–C″) Accumulation of AN-tdTomato in root tip (B and B′) and globular-stage embryo (C). (B) shows an overview and (B′) a higher magnification. (C) shows a high magnification confocal image, (C′) a bright field image, and (C″) a confocal overview image. Color scale in (B′) indicates false colors used (low, left; high, right). (D–E″) Co-localization of AN-tdTomato and SOK1-YFP in RPS5A-SOK1-YFP roots in control (D) or mannitol-treated (E) conditions. (F) Co-localization of AN-tdTomato and SOK1ΔDIX-YFP in RPS5A-SOK1ΔDIX -YFP roots in control conditions. (D′), (E′), and (F′) show AN-tdTomato localization and (D″), (E″), and (F″) show SOK1-YFP or SOK1ΔDIX-YFP in the same region (dashed boxes). Scale bars, 10 μm (B, D–F); 5 μm (C). See also Figure S4.

The effector proteins through which SOSEKI proteins carry out their function are unknown, and the SOSEKI domains (Figure 2A) do not provide any clues as to their identities. To identify SOSEKI effectors, we used pull-downs and mass spectrometry on roots from *Arabidopsis* seedlings in which SOK1-YFP was overexpressed from the *RPS5A* promoter. Pull-downs from the pSOK1-SOK1-YFP line did not retrieve SOK1 proteins, likely because of low protein abundance (Figure S4A). AtSOK1 was affinity-purified from RPS5A-SOK1-YFP roots, in addition to AtSOK4 (presumably reflecting hetero-polymerization with SOK1-YFP) (Figure 6A). Interestingly, SOK1-YFP pull-down also recovered ANGUSTIFOLIA (AN) (Figure 6A), the plant ortholog of mammalian C-terminal binding protein (CtBP). CtBP is an NAD/NADH-binding metabolic sensor (Fjeld et al., 2003) that is recruited into various multiprotein complexes, including transcriptional repressor complexes (Chinnadurai, 2002), and adenomatous polyposis coli, a component of the Axin degradosome (Hamada and Bienz, 2004). The molecular function of *Arabidopsis* ANGUSTIFOLIA remains poorly defined, a mutation in *an* causes defects in cell shape, cell division orientation, and organ shape (Bai et al., 2013, Tsuge et al., 1996). Affinity purification of SOK2-YFP and SOK3-YFP likewise recovered each tagged SOK protein, as well as other SOK proteins and ANGUSTIFOLIA (Figures S4B and S4C). ANGUSTIFOLIA thus seemed an excellent candidate for an effector of SOSEKI in orienting cell division.

To investigate ANGUSTIFOLIA localization, we generated a line in which ANGUSTIFOLIA was C-terminally fused to tdTomato, and driven from the endogenous *ANGUSTIFOLIA* promoter (AN-tdTomato). This fusion protein is functional, as it complements the characteristic two-branched trichome defects in *an-1* mutant leaves (Figures S4D–S4F). We observed diffuse fluorescent signals from AN-tdTomato throughout root cells, in addition to clear enrichment near cell edges (Figure 6B, arrowheads). When analyzed in the embryo, membrane-associated AN-tdTomato puncta could be clearly distinguished (Figure 6C).

AN could either act as a regulator of SOK localization, or it may represent an effector whose localization depends on SOK proteins. To determine if SOK1-localization requires the AN protein, we localized SOK1-YFP (expressed from the *RPS5A* promoter) in an *an* loss of function mutant. Neither membrane association nor polar localization of SOK1-YFP protein was obviously altered in the *an* mutant (Figures S4G and S4H), suggesting that AN is not a regulator of SOK1 localization.

Conversely, when introduced into RPS5A-SOK1-YFP lines, some of the AN-tdTomato signals accumulate at the cell edges marked by SOK1-YFP (Figure 6D; see Figure S4I for negative control), likely owing to recruitment by the misexpressed SOK1-YFP. To test the dependence of AN-tdTomato localization on SOK1 more directly, we treated roots with mannitol, displacing SOK1 from the polar membrane edge (Yoshida et al., 2019). When imaged soon after mannitol treatment, SOK1 was displaced from the polar edge in some, but not all cells (Figure 6E). Co-localizing AN-tdTomato with SOK1-YFP under these conditions showed strong correlation between AN-tdTomato and SOK1-YFP localization (Figure 6E), revealing an intimate interaction between them.

To determine whether the polar AN-tdTomato localization requires DIX-dependent SOK1 polymerization, we examined AN-tdTomato in lines expressing SOK1-YFP without its DIX domain (RPS5A-SOK1-ΔDIX-YFP) (Yoshida et al., 2019), whose SOK1-YFP is delocalized from the cell edges (Figure 6F). In these lines, AN-tdTomato no longer co-localized with SOK1-YFP (Figures 6F and S4I). Thus, the DIX-dependent polymerization of AtSOK1 appears to be required for ANGUSTIFOLIA recruitment, reminiscent of Dishevelled, whose DIX-dependent polymerization mediates recruitment of its Axin effector into Wnt signalosomes by increasing its avidity for this effector. This suggests a deep functional analogy regarding the assembly of protein condensates by plant SOSEKI and animal Dishevelled proteins.

### The DIX Domain Can Be Traced Back to Basal Eukaryotes

**Figure 7:**
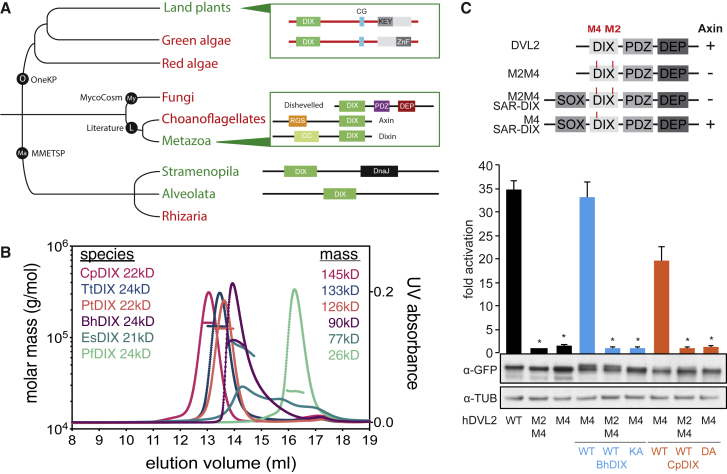
The DIX Domain Can Be Traced to Unicellular Eukaryotes (A) Extended phylogeny of DIX proteins across eukaryotic kingdoms; black, sources of data (O, OneKP; My, MycoCosm; Ma, MMETSP; L, literature); boxes, domain topology. Green branches mark groups with DIX domain-containing proteins, and red branches without DIX domain-containing proteins. (B) SEC-MALS of purified DIX domains from *Cryptosporidium parvum* (CpDIX), *Tetrahymena thermophila* (TtDIX), *Paramecium tetraurelia* (PtDIX), *Blastocystis hominis* (BhDIX), *Ectocarpus siliculosus* (EsDIX), or *Plasmodium falciparum* (PfDIX); curves and line traces as in main Figure 3A. (C) SuperTOP reporter assays as in main Figure 5E. DIX domains in chimeras are from *Blastocystis hominis* (BhDIX) or *Cryptosporidium parvum* (CpDIX); data represent the mean ± SEM (n = 6); ^∗^p < 0.0001 (one-way ANOVA) for comparison to respective WT DIX domain. Underneath: corresponding western blots indicating expression levels (TUB, β-tubulin). See also Figure S5 and Table S3.

**Figure S5:**
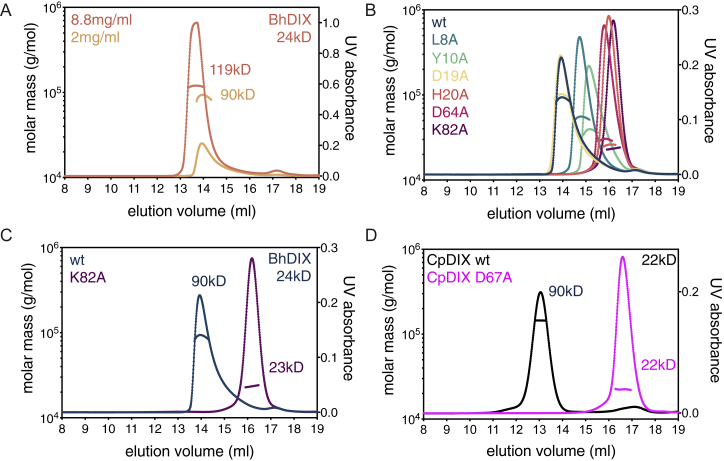
Biochemical Characterization of SAR Group DIX Domains, Related to Figure 7 (A–D) SEC-MALS of purified wild-type (A) and various mutant (B and C) DIX domains from *Blastocystis hominis* (BhDIX) or wild-type and mutant DIX domains from (D) *Cryptosporidium parvum* (CpDIX) (D); curves and line traces as in main Figure 3A.

We wondered whether we could identify DIX domain-containing proteins in other eukaryotic lineages, by extending our phylogenetic analysis beyond animals and plants. We used broad fungal genomes (MycoCosm) (Grigoriev et al., 2014) and unicellular eukaryote transcriptome datasets (MMETSP) (Keeling et al., 2014) to search for DIX-like sequences, using AtSOK1-5, MpSOK, and DVL2 DIX domains as queries. While our searches of the fungal kingdom were negative, we readily identified DIX-like sequences in SAR (Stramenopiles, Alveolates, Rhizaria) group organisms (Figure 7A), including the human parasites *Plasmodium*, *Blastocystis*, and *Cryptosporidium*, but also the plant pathogen *Phytophthora*, as well as brown algae and the ciliates *Paramecium* and *Tetrahymena*. However, many SAR group species lack DIX-like domains, as indicated by comprehensive searches of all fully sequenced and annotated genomes of SAR group organisms. Importantly, none of the DIX-like sequences in SAR group organisms are associated with any of the conserved domains found in plant SOSEKI or Dishevelled (Figure 7A), suggesting that they may operate in distinct functional contexts. However, SAR group DIX domains are clearly capable of polymerization, as indicated by SEC-MALS analysis of purified DIX domains from *Ectocarpus siliculosus* (brown alga), *Cryptosporidium parvum* (apicomplexan), *Paramecium tetraurelia* (ciliate), and three other species (Figure 7B). Furthermore, the *in vitro* polymerization of these DIX domains depends on their concentration (Figure S5A) and is abrogated by mutations in conserved residues of their putative DIX polymerization interfaces (Figures S5B–S5D). Thus, SAR DIX domains are capable of undergoing concentration-dependent head-to-tail polymerization like their animal and plant counterparts.

To determine whether these SAR DIX domains can also complement DVL2 DIX, we used the DIX substitution assays as described above (Figure 5), but replacing DVL2 DIX with SAR DIX, monitoring DIX-dependent puncta formation, and signaling to β-catenin. Indeed, WT, but not polymerization-defective DIX domains, from *Blastocystis* and *Cryptosporidium* can fully substitute for DVL2 DIX in terms of β-catenin signaling (Figures 7C, S5C, and S5D). Thus, SOSEKI DIX and SAR DIX domains are functionally equivalent to DVL2 DIX regarding their biochemical properties and signaling activity, suggesting that the SAR DIX domains reflect the ancestral version of animal and plant DIX domains.

## Discussion

Cell polarity is essential for the development of multicellular life. Proteins establishing polarity and their pathways have been studied in detail in animals, but there is limited knowledge about polarity formation in plants. In this latter kingdom, the translation of pre-mitotic cell polarity to the orientation of cell division is of fundamental importance because the rigid cell wall prevents movement or reorientation of the cell. Although the components of polarity signaling pathways in animals such as Wnt-Dishevelled are missing from plant genomes (Kania et al., 2014), we recently discovered the *Arabidopsis* SOSEKI proteins as polarly localized proteins (Yoshida et al., 2019). Here, we establish that SOSEKI proteins are highly conserved in all land plants and likely represent components of an ancient polarity system. Furthermore, we show that they contain a bona fide DIX domain whose polymerization is essential for their localization and cellular function. This domain is structurally and functionally equivalent to the DIX domain of Dishevelled and is required for the polar recruitment of the effector protein ANGUSTIFOLIA. Thus, the paradigm of dynamic protein polymerization conferring high local concentrations, initially derived from the study of the polarity protein Dishevelled (Bienz, 2014, Gammons and Bienz, 2018), also underlies cell polarity in plants.

### Land Plants Have a Common Polarity System

Various polarly localized proteins have been described in plants, decorating unique membrane domains in different cell types (Kania et al., 2014). Based on studies that mostly used PIN auxin hormone transport proteins and Boron transporters BOR1 and NIP5;1 (Geldner et al., 2001, Kania et al., 2014, Takano et al., 2010), a model has emerged in which exocytosis and endocytosis are balanced to maintain polar protein localization. Furthermore, studies on Rop GTPases identified a dynamic mechanism for local outgrowth in various cell types, based on local effects on the cytoskeleton (Yang and Lavagi, 2012). Given that much of this research was performed in a single species—the flowering plant *Arabidopsis thaliana*—a largely unexplored question is to what degree polarity systems are universal among land plants. Localization of PIN proteins in the moss *Physcomitrella* revealed bi-polar localization in leaf cells and tip localization in tip-growing cells, distinct from the polarity patterns of orthologous *Arabidopsis* PIN proteins (Gälweiler et al., 1998, Geldner et al., 2001, Viaene et al., 2014).

Using phylogenomic analysis, we found that all land plants for which genome sequence information is available have SOSEKI proteins, defined by common conserved elements that are relevant for their localization and activity in *Arabidopsis*. In addition, localization of *Physcomitrella* and *Marchantia* SOSEKI proteins show distinct edge polarity coordinated among cells, highly reminiscent of the polar localization of SOSEKI proteins in *Arabidopsis*. This suggests that SOSEKI proteins are ancient polarity proteins that reflect a common polarity reference system in all land plants. We note that their biological function has so far been deduced from overexpression studies in *Arabidopsis* and thus requires confirmation by loss-of-function genetics. However, the similar patterns and stability of their polar localizations in *Arabidopsis* (Yoshida et al., 2019) and *Physcomitrella* and *Marchantia* (Figures 1C–1G) suggest a fundamental role of SOSEKI proteins as polarity landmarks or readouts. Indeed, SOSEKI proteins can only be delocalized by osmotic or mechanical manipulations affecting the cell wall (Yoshida et al., 2019), and their polar localizations are inert and virtually refractory to experimental perturbation. In contrast, most other *Arabidopsis* proteins previously implicated in cell polarity show highly dynamic polar localization that is readily perturbed by membrane trafficking or cytoskeletal remodeling (Geldner et al., 2001, Kania et al., 2014, Takano et al., 2010). Therefore, based on their deep conservation and robust localization patterns, SOSEKI proteins are excellent candidates for components of signaling pathways that determine planar cell polarity.

### A Conserved Biochemical Paradigm Underlies Cell Polarity

By determining the crystal structure of AtSOK4 DIX, we established that this domain is the closest known relative of the DVL2 DIX domain. Our evidence indicates that the polymerization of this domain is required for polar localization of SOSEKI proteins in *Arabidopsis*. Furthermore, the ability of this domain to substitute for the DVL2 DIX domain in a cross-kingdom complementation assay indicates their functional equivalence. Dishevelled depends on DIX-dependent polymerization to attain a high local concentration, to acquire the high avidity needed to recruit its low-affinity signaling effector Axin to the Wnt signalosome (Gammons and Bienz, 2018). Similarly, *Arabidopsis* SOK1 protein depends on its DIX domain for recruitment of ANGUSTIFOLIA, a putative SOSEKI effector, to a polar membrane domain. It thus appears that the same paradigm operates in animal and plants for assembly of protein condensates with high avidity for effector proteins.

### Polar Domain Formation Depends on DIX-Dependent Polymerization

Cell polarity requires the definition of cellular subdomains, often at the plasma membrane. Lateral diffusion along the membrane is highly unfavorable to maintaining polar localization and must therefore be limited. This could be achieved by physical barriers, such as tight junctions in animals (Shin et al., 2006) and cell wall modifications in plants (Lee et al., 2013, Martinière et al., 2012). However, physical diffusion barriers will act as generic limitations to the diffusion of all membrane-associated proteins and are therefore useful only in cells with long-term fixed organization. It is difficult to imagine how such barriers would be compatible with flexible development in dividing cells whose architecture is fluid.

Polymerization of polarity proteins may provide an alternative mechanism to limit their diffusion. Indeed, mutating the DIX domain of SOK1 leads to its diffusion over a much wider membrane domain and its delocalization from the polar edge. This suggests that cell polarity is achieved in two steps: recruitment of SOSEKI proteins to a wide membrane domain, followed by their polymerization, which tightens their localization to a polar patch. Visualization of early stages of SOSEKI polarization suggests that this second step may in fact include a stage at which membrane-associated puncta coalesce into broad patches, that are tightened to sharp edge localization. Polymerization of SOSEKI proteins may therefore serve a dual function, namely (1) limiting diffusion to achieve tight polar localization, and (2) generating a high local protein concentration to attain a high avidity for low-affinity effector proteins. Notably, the latter is contingent on the former, which ensures that recruitment of effectors occurs subsequent to SOSEKI protein polymerization at the membrane, rather than at the lower SOSEKI protein concentration in the cytosol. Polymerization therefore provides an elegant mechanism to orchestrate the polar localization of polarity-determining proteins and the subsequent recruitment of their ligands that effect cell polarity.

Notably, several polarity proteins in animals such as Par proteins and atypical protein kinase C bear a PB1 domain (Hirano et al., 2005), a structural relative of the DIX domain, and evidence suggests that this domain also engages in head-to-tail polymerization (Bienz, 2014). Therefore, these PB1 domains, like the SOSEKI DIX domains, may have dual functions in conferring both polar localization and high local concentration for effector recruitment via polymerization.

### The DIX Domain Has an Ancient Origin

The DIX domain was thought to be limited to Dishevelled and Axin in the Wnt signaling pathway. However, our study led to the discovery of bona fide DIX domains in plants and unicellular SAR group eukaryotes. Intriguingly, other eukaryotic kingdoms such as fungi and green algae do not encode any DIX domains. Furthermore, these domains are only present in a subset of SAR group organisms. The DIX domain may have originated in basal eukaryotes whereby only some SAR organisms retained the ancestral domain, evolving separately into plant and animal DIX domains. Alternatively, the domain originated in SAR organisms, and was passed on to plants and animals by horizontal gene transfer. In support of the latter, several SAR group organisms such as *Phytophthora* and *Cryptosporidium* are parasites or pathogens of plant or animals that potentially engage in horizontal gene transfer. In either scenario, it appears that plants and animals have independently evolved an ancestral DIX domain from basal eukaryotes to adapt it to their own polarity systems.

## STAR★Methods

### Key Resources Table

**Table:** 

REAGENT or RESOURCE	SOURCE	IDENTIFIER
**Antibodies**		

α-GFP (rabbit)	Sigma-Aldrich	Cat#G1544; RRID: AB_439690
α-Flag (mouse)	Sigma-Aldrich	Cat#F1804; RRID: AB_262044
α-β-tubulin (mouse)	Sigma-Aldrich	Cat#T4026; RRID: AB_477577
HRP conjugated Goat α-Rabbit	Santa Cruz Biotechnology	Cat#sc-2301; RRID: AB_650500
HRP conjugated Goat α-Mouse	Santa Cruz Biotechnology	Cat#sc-2005; RRID: AB_631736
Alexa Fluor 488 conjugated Goat α-Rabbit	Life Technologies	Cat#A11008; RRID: AB_143165
Alexa Fluor 546 conjugated Goat α-Mouse	Life Technologies	Cat#A11003; RRID: AB_141370
MultiMACS GFP isolation kit	Miltenyi Biotec	130-094-252

**Chemicals, Peptides, and Recombinant Proteins**		

Ni-NTA Agarose	QIAGEN	Cat#30210
Polyethylenimine, linear, MW25000	Polysciences	Cat#23966
EDTA-free Protease Inhibitor Cocktail	Roche	Cat#04693159001
VectaShield with DAPI	Vector Laboratories	Cat#H-1200
Isopropyl-β-D-1thiogalactopyranoside, IPTG	Sigma-Aldrich	Cat#I6758
Imidazole	Sigma-Aldrich	Cat#56748
KOD DNA polymerase	Merck Millipore	Cat#71086-4
DNase I	Sigma-Aldrich	Cat#D4527
ECL Western Blotting Detection Reagent	Amersham	Cat#RPN2106
LiChroprep RP-18	Merck	Cat#109303
Acetonitrile HPLC-S Gradient Grade	Biosolve Chimie SARL	Cat#00012007
NP-40	AppliChem	Cat#A1694
Trypsin sequencing grade	Roche	Cat#11047841001
Iodoacetamide Bio-Ultra	Sigma-Aldrich	Cat#I1149
Propidium Iodide	Sigma-Aldrich	Cat#P4170

**Critical Commercial Assays**		

Dual-Luciferase Reporter Assay System	Promega	Cat#E1910
HiPure Plasmid Midiprep Kit	Invitrogen	Cat#K210005

**Deposited Data**		

SOK4 PDB file	This paper	https://www.rcsb.org/structure/6RSN
Plant SOK phylogeny	This paper	https://itol.embl.de/shared/dolfweijers

**Experimental Models: Cell Lines**		

HEK293T	ATCC	Cat#CRL-3216
COS-7	ATCC	Cat#CRL-1651
Dvl TKO	Gammons et al., 2016b	N/A

**Experimental Models: Organisms/Strains**		

*Arabidopsis thaliana* accession Col-0	Standard accession	N/A
*Arabidopsis thaliana* mutant *an-1*	Tsukaya et al., 1994	N/A
*Physcomitrella patens* P_EF1α_::mCherry-α-tubulin	Kosetsu et al., 2017	N/A
*P. patens* P_EF1α_::mCherry-α-tubulin / PpSOK1-Citrine	This paper	N/A
*P. patens* P_EF1α_::mCherry-α-tubulin / PpSOK2-Citrine	This paper	N/A
*P. patens* P_EF1α_::mCherry-α-tubulin / PpSOK3-Citrine	This paper	N/A
*P. patens* P_EF1α_::mCherry-α-tubulin / PpSOK4-Citrine	This paper	N/A
*M. polymorpha 35S::mCitrine-MpSOK*	This paper	N/A
*Arabidopsis thaliana* Col-0 pSOK1::SOK1-YFP	Yoshida et al., 2019	N/A
*Arabidopsis thaliana* Col-0 pRPS5A::SOK1-YFP	Yoshida et al., 2019	N/A
*Arabidopsis thaliana* Col-0 pRPS5A::SOK2-YFP	Yoshida et al., 2019	N/A
*Arabidopsis thaliana* Col-0 pRPS5A::SOK3-YFP	Yoshida et al., 2019	N/A
*Arabidopsis thaliana* Col-0 pRPS5A::SOK1 lacking DIX-YFP	Yoshida et al., 2019	N/A
*Arabidopsis thaliana* Col-0 pRPS5A::SOK1 C233A-YFP	This paper	N/A
*Arabidopsis thaliana* Col-0 pRPS5A::SOK1 C233A/G234A -YFP	This paper	N/A
*Arabidopsis thaliana* Col-0 pRPS5A:: SOK1 C307A/C310A-YFP	This paper	N/A
*Arabidopsis thaliana* Col-0 pRPS5A::SOK5 C303A-YFP	This paper	N/A
*Arabidopsis thaliana* Col-0 pRPS5A::SOK5 E356Q/E359Q-YFP	This paper	N/A
*Arabidopsis thaliana* Col-0 pRPS5A::SOK1 H29D/D78A-YFP	This paper	N/A
*Arabidopsis thaliana* Col-0 pRPS5A::SOK1 H29D/D78R-YFP	This paper	N/A
*Arabidopsis thaliana* Col-0 pRPS5A:: hDvl2DIX SOK1-YFP	This paper	N/A
*Arabidopsis thaliana* Col-0 pAN::AN-TdTomato	This paper	N/A
*Arabidopsis thaliana an-1* pAN::AN-TdTomato	This paper	N/A
*Arabidopsis thaliana* Col-0 pAN::AN-TdTomato and pRPS5A::SOK1-YFP	This paper	N/A
*Arabidopsis thaliana* Col-0 pAN::AN-TdTomato and pRPS5A::SOK1 lacking DIX-YFP	This paper	N/A

**Oligonucleotides**		

All oligonucleotides are listed in Table S4	This paper	N/A

**Recombinant DNA**		

Plasmid: pCMV-tag2b-Axin	Fiedler et al., 2011	N/A
Plasmid: pEGFP-DVL2	Fiedler et al., 2011	N/A
Plasmid: pTA-Luc m50 Super 8x TopFLASH	Veeman et al., 2003	Addgene Cat#12456
Plasmid: pRL-CMV Renilla luciferase	Promega	Cat#E2261
Plasmid: pCTRN-nptII	Hiwatashi et al., 2008	GenBank AB697058
Plasmid: pCTRN-nptII-PpSOK1-RF+LF	This paper	N/A
Plasmid: pCTRN-nptII-PpSOK2-RF+LF	This paper	N/A
Plasmid: pCTRN-nptII-PpSOK3-RF+LF	This paper	N/A
Plasmid: pCTRN-nptII-PpSOK4-RF+LF	This paper	N/A
Plasmid: pRPS5A::AtSOK1-YFP	Yoshida et al., 2019	N/A
Plasmid: pRPS5A::AtSOK2-YFP	Yoshida et al., 2019	N/A
Plasmid: pRPS5A::AtSOK3-YFP	Yoshida et al., 2019	N/A
Plasmid: pRPS5A::AtSOK1 lacking DIX-YFP	Yoshida et al., 2019	N/A
Plasmid: pRPS5A::AtSOK1 C233A-YFP	This paper	N/A
Plasmid: pRPS5A::AtSOK1 C233A/G234A -YFP	This paper	N/A
Plasmid: pRPS5A:: AtSOK1 C307A/C310A-YFP	This paper	N/A
Plasmid: pRPS5A::AtSOK5 C303A-YFP	This paper	N/A
Plasmid: pRPS5A::AtSOK5 E356Q/E359Q-YFP	This paper	N/A
Plasmid: pRPS5A::AtSOK1 H29D/D78A-YFP	This paper	N/A
Plasmid: pRPS5A::AtSOK1 H29D/D78R-YFP	This paper	N/A
Plasmid: pRPS5A:: DvlDIX AtSOK1-YFP	This paper	N/A
Plasmid: pAN::AN-TdTOMATO	This paper	N/A
Plasmid: pPLV28	De Rybel et al., 2011	N/A
Plasmid: pPLV23	De Rybel et al., 2011	N/A
Plasmid: 35S-mCitrine-MpSOSEKI	This paper	N/A

**Software and Algorithms**		

Phylogenetic Analysis pipeline	Mutte et al., 2018	N/A
MAFFT v7 (ver7.123b)	National Institute of Advanced Industrial Science and Technology (Japan)	https://mafft.cbrc.jp/alignment/server/
Phyutility v2.2.6	Smith and Dunn, 2008	https://code.google.com/archive/p/phyutility/downloads
tBLASTn module at JGI MycoCosm	Joint Genome Institute (USA)	https://mycocosm.jgi.doe.gov/fungi
RAxML v8.1.20	Stamatakis, 2014	https://github.com/stamatak/standard-RAxML
PartitionFinder2	Lanfear et al., 2017	https://github.com/brettc/partitionfinder/releases/tag/v2.1.1
MEME motif discovery program (ver 4.12.0)	Bailey et al., 2009	http://meme-suite.org/doc/download.html
MaxQuant software package v1.6.8.0	Tyanova et al., 2016a	https://www.maxquant.org
Perseus v1.6.2.3.	Tyanova et al., 2016b	https://www.maxquant.org
R	R Foundation	https://www.r-project.org/

**Other**		

OneKP consortium database	Matasci et al., 2014	http://sites.google.com/a/ualberta.ca/onekp/
Phytozome ver11	Joint Genome Institute (USA)	https://phytozome.jgi.doe.gov/pz/portal.html
MMETSP data	Keeling et al., 2014	N/A

### Lead Contact and Materials Availability

Requests for further information or reagents should be directed to the lead contact, Dolf Weijers (dolf.weijers@wur.nl).

There are/are no restrictions on the availability of materials and reagents mentioned in this work.

### Experimental Model and Subject Details

#### Plant model: *Arabidopsis thaliana*

For all experiments on *Arabidopsis thaliana* the Col-0 ecotype was used. The *an-1* mutant line is also in Col-0 background and was described (Tsukaya et al., 1994). Identity of this mutant line was confirmed by observing trichome and leaf morphology defects. The wild-type pSOK1::SOK1-YFP, pRPS5a::SOK-YFP lines and the pRPS5a::SOK1 lacking DIX domain-YFP have previously been described (Yoshida et al., 2019). Mutant pRPS5a::SOK-YFP lines have been generated for this study, see Method Details section below.

Seeds were sterilized in 25% thin bleach (local supermarket brand, < 5% sodium hypochlorite), 75% ethanol for 8 minutes, washed twice in 70% ethanol and once in 96% ethanol. After drying, seeds were plated on half strength Murashige and Skoog (½MS) with 10 g/l sucrose and supplemented with 15mg/l Phosphinothricin or 50 mg/l Kanamycin if selection was required. The seeds were stratified at 4°C for 1-2 days before transferring the plates to the growth room. Plants were cultured at 22°C, 75% humidity under long-day conditions (16h light, 8h dark). Seedlings were imaged or harvested for IP-MS/MS at 5-6 days after transfer to the growth room. To obtain mature plants, seedlings were transferred to soil and continued to grow under the conditions described.

#### Plant model: *Physcomitrella patens*

All *Physcomitrella patens* (ecotype ‘Gransden’) (Engel, 1968, Ashton and Cove, 1977) strains expressing PpSOK-Citrine fusions used in this study were generated in a background line expressing mCherry-α-tubulin driven from the PpEF1α promoter (Kosetsu et al., 2017). Plants were grown on BCDAT medium under continuous light at 25°C until transformation. Initial transformants were selected on BCDAT plates supplemented with 20 mg/l G418. After three weeks of release from selection, plants were reinoculated on G418-containing medium to select for stable transformants. Transformed plants were grown under the same temperature and light conditions as *Arabidopsis*, as described above. For propagation, plants were homogenized with a razor blade, and the fragments were plated on fresh BCD plates. To observe naturally occurring buds, plants were grown for 3-5 weeks until enough buds had formed for observation. To enhance rates of 3D bud production, plants were first homogenized and grown on BCD medium overlaid with cellophane for one week. Then, the cellophane containing the plants was transferred to new BCD plates supplemented with 1μM 6-Benzylaminopurine (BAP) and grown for 2-10 days until the desired stage of bud formation was reached.

#### Plant model: *Marchantia polymorpha*

Male and female accessions of *Marchantia polymorpha,* Takaragaike-1 (Tak-1) and Tak2, respectively were used and maintained asexually. *Marchantia* was grown on half strength Gamborg’s B5 medium containing 0.5g/l MES and 10 g/l agar (Gamborg et al., 1968). Plants were kept under 50-60 μmol photons per m^-2^s^-1^ continuous white light at 22°C. Spores generated by crossing Tak-1 and Tak-2 were used for transformation to generate the 35S-mCitrine-MpSOSEKI transgenic line.

#### Cell cultures: HEK293T and COS-7

HEK293T and COS-7 cells were cultured in DMEM (GIBCO), supplemented with 10% fetal bovine serum (FBS) and penicillin/streptomycin at 37°C in a humidified atmosphere with 5% CO_2_. All cells were screened regularly for mycoplasma. Transient transfections of cells were performed using polyethylenimine (PEI, Polysciences) in DMEM with 10% FBS.

### Method Details

#### Generation of transgenics

For transformation of *Arabidopsis*, floral dipping was performed as described (Clough and Bent, 1998). *Agrobacterium tumefaciens* strain GV3101 harboring helper plasmid pSOUP was transformed by electroporation with plasmids containing the relevant mutant SOK or AN versions. After two days of selection at 28°C on LB agar supplemented with 25mg/l rifampicin, 50mg/l gentamycin, 2mg/l tetracycline and 50mg/l kanamycin, colonies were picked and cultured overnight at 28°C in liquid LB plus aforementioned antibiotics. The following morning, 2.5g sucrose and 25μl Silwet L-77 were added to 50ml bacterial culture. *Arabidopsis* flowers were dipped into this suspension and incubated in a closed box overnight. Next day, the plants were put upright and cultured under long-day conditions as described above.

*Physcomitrella* was transformed as follows: Approximately 30 μg of plasmid DNA harboring the gene targeting constructs for Citrine-tagging of PpSOKs was linearized and transformed into a pEF1α::mCherry-tubulin *P. patens* line (Kosetsu et al., 2017). Transformation was performed using protoplasts generated with Driselase and the PEG/heat-shock-based method described by Nishiyama et al., (2000). Successful integration of the Citrine tag at the C terminus of the endogenous PpSOK genes through homologous recombination was confirmed by PCR using oligonucleotides listed in Table S4.

*Marchantia polymorpha* sporelings were transformed with *Agrobacterium* harboring 35S-mCitrine-MpSOSEKI according to an established method (Ishizaki et al., 2008). The transformed sporelings were plated and grown on half-strength Gamborg’s B5 medium supplemented with 10μg/ml hygromycin and 100μg/ml cefotaxime.

#### Generation of plasmids

Primers used in this study are described in Table S4. Mutations in *Arabidopsis* SOK1 and SOK5 fusion proteins were generated by overlap extension PCR: *SOK* was amplified in two parts from a SOK-YFP cDNA-containing plasmid, with primers containing the desired mutation. Both fragments were fused together and cloned into a pGIIB pRPS5a::LIC-NOSt (pPLV28) vector (De Rybel et al., 2011) using SLICE (Zhang et al., 2012). pAN:AN-TdTOM was generated by cloning a ∼2kb fragment amplified from the promoter and the protein coding region from *Arabidopsis thaliana* genomic DNA into pPLV23 (De Rybel et al., 2011) with SLICE cloning (Zhang et al., 2012). All plasmids were verified by sequencing.

For homologous recombination in *Physcomitrella*, ∼1kb of the genomic region upstream and downstream of the predicted stop codon was amplified by PCR and cloned into pCTRN-nptII

(Hiwatashi et al., 2008) using the restriction sites in Table S4. All constructs were verified by sequencing.

To generate 35S-mCitrine-MpSOSEKI, the genomic region spanning the entire coding sequence of the MpSOSEKI gene was amplified using primers CA74-MpSOSEKI fw cacc and CA75-MpSOSEKI rv stop. The amplified fragment was cloned into the pENTER/D-TOPO vector using the Gateway TOPO cloning kit (Life technologies). This entry clone was recombined into the pMpGWB105 (Ishizaki et al., 2015) by the LR Clonase II (Life technologies) according to the manufacturer’s recommendations.

DIX sequences (Table S5) for *in vitro* and cell-based assays were generated by gene synthesis (gBlocks, IDT), amplified by PCR and cloned into mammalian and bacterial expression vectors by restriction free cloning. Mutagenesis was carried out with standard PCR-based methods, using KOD DNA polymerase (Merck Millipore) and clones were verified by sequencing.

#### Phylogenetic analysis

Nucleotide and protein sequences of each SOSEKI ortholog from *Marchantia polymorpha*, *Physcomitrella patens*, *Amborella trichopoda*, *Oryza sativa*, *Zea mays*, *Solanum lycopersicum* and *Arabidopsis thaliana* were obtained from Phytozome ver11 (https://phytozome.jgi.doe.gov/pz/portal.html). Various DIX domain-containing proteins were obtained from UniProt and GenBank databases (for IDs, see Table S3). The tBLASTn module at JGI MycoCosm (https://mycocosm.jgi.doe.gov/fungi) was used to search for DIX domain-containing proteins in fungi with plant (*A. thaliana*) and animal (*H. sapiens*) DIX domains as query sequences. Data access to > 1000 plant transcriptomes was provided by the OneKP consortium (http://sites.google.com/a/ualberta.ca/onekp/) (Matasci et al., 2014). A representative set of transcriptomes (see Mutte et al., 2018) for each taxonomic clade (level: order) was used to search for SOSEKI proteins in OneKP plant transcriptomes. To determine the presence and evolution of SOSEKI or DIX domain-containing proteins in the SAR group, the Marine Microbial Eukaryotic Transcriptome Sequencing Project (MMETSP) data (Keeling et al., 2014) was used, and genes were identified as described for OneKP data. Phylogenetic analysis was performed with the pipeline developed by Mutte et al. (2018). Briefly, all collected nucleotide sequences from various species were aligned with MAFFT v7 (ver7.123b; https://mafft.cbrc.jp/alignment/server/), and alignment positions with > 70% gaps were removed using Phyutility v2.2.6 (Smith and Dunn, 2008). Furthermore, a phylogenetic tree was constructed using RAxML v8.1.20 (Stamatakis, 2014) with the GTR model of evolution, identified with PartitionFinder2 (Lanfear et al., 2017). Phylogenetic trees were visualized in iTOL and can be accessed at https://itol.embl.de/shared/dolfweijers.

#### Identification of domains and motifs in SOSEKI proteins

Protein sequences of the transcripts used in phylogenetic tree construction were used for domain finding using the MEME motif discovery program with additional parameters “*-mod zoops -nmotifs 15 -minw 10*” (ver 4.12.0) (Bailey et al., 2009). Among 15 elements identified, 4 spanned the N-terminal most 100 residues, and were identified as DIX domains. Motifs that were specific to a certain clade or motifs that did not show conservation of significant amino acids were discarded.

#### SuperTOP assays

For luciferase reporter assays, Dishevelled null mutant HEK293T cells (Gammons et al., 2016b) were transfected with SuperTOP (Veeman et al., 2003) and CMV-Renilla control plasmids with PEI. 22 hours post-transfection, cells were lysed for 20 minutes in Über buffer (20 mM Tris pH 7.4, 10% v/v glycerol, 200 mM NaCl, 1 mM EDTA, 5 mM NaF, 2 mM Na_3_OV_4_, 0.2% Triton X-100 and Protease Inhibitor cocktail tablets (Roche)). Lysates were cleared by centrifugation and analyzed with the DualGlo Luciferase Reporter Assay kit (Promega) according to the manufacturer’s protocol using 10 μl lysate with 50 μl reagents in an opaque 96-well plate. Measurements were made with an Orion Microplate Luminometer (Berthold). Values were normalized to Renilla luciferase, and are shown as mean ± SEM relative to the signaling-incompetent DVL2-M2M4 mutant (set to 1 in Figures 5A and 7D). Experiments were repeated 6 times.

An aliquot of each reaction was resolved by SDS-PAGE and analyzed by western blotting using primary antibodies against GFP and β-tubulin, followed by HRP-conjugated secondary antibodies against rabbit or mouse, respectively. Proteins were detected with ECL Western Blotting Detection Reagent (Amersham) and developed on film.

#### Protein expression and purification

6xHisLip- or 6xHisBRIL-tagged recombinant proteins were purified from BL21(DE3) pRARE2 *E. coli* bacterial strains. Bacteria were grown in LB media supplemented with appropriate antibiotic to OD_600_ 0.6, then dropped to a lower temperature (16 to 24°C) and induced at OD_600_ 0.8 by addition of 0.4 mM isopropyl β-D-1-thiogalactopyranoside (IPTG). Proteins were expressed for 3 hours or over night. Cell pellets were resuspended in lysis buffer (25 mM Tris-HCl pH 8, 200 mM NaCl, 20 mM imidazole, 10 μg/ml DNase, protease inhibitor cocktail) and lysed by high-pressure homogenization with an Emulsiflex C-3 (Avestin). Lysates were cleared by ultracentrifugation (140,000x *g*, 30 minutes, 4°C) and mixed with Ni-NTA agarose. Beads were washed multiple times with lysis buffer, and 6xHistagged protein was eluted with lysis buffer supplemented with 500 mM imidazole. Each protein was purified by size exclusion chromatography, and protein purity was assessed by SDS-PAGE.

Selenomethionine labeled samples were expressed in M9 minimal medium supplemented with 0.4% glucose, antibiotics, trace elements and 30 mL overnight culture per liter expression culture. Cultures were grown at 37°C to OD_600_ 0.6, at which point individual amino acids (0.4 g/l lysine, threonine, phenylalanine and 0.2 g/l leucine, isoleucine, valine and selenomethionine) were added. Cells were induced at OD_600_ 0.8 with IPTG and processed essentially as described above.

#### Protein crystallization and data collection

6xHisLip-TEV-SOK4 bearing D85A (which blocks polymerization) was cleaved by TEV protease (protein:TEV ratio 80:1) overnight at 4°C. The tag was removed by binding SOK4 protein to a HiTrapSP column (GE Healthcare) with a linear NaCl gradient (0-1 M NaCl) in 25 mM BIS-TRIS pH 6.5, 0.06% NaN_3_. Eluted fractions were separated on a HiLoad 26/600 Superdex 75 pg column (GE Healthcare) in 25 mM BIS-TRIS pH 6.5, 150 mM NaCl. Pure fractions of SOK4 D85A were concentrated with a 3 kD MWCO Vivaspin 20 concentrator (Sartorius) to 9 mg/mL. Prior to crystallization, 1 mM TCEP was added and samples were cleared by centrifugation for 15 minutes at 100,000 *rcf.* Crystallization trials with multiple commercial crystallization kits were performed in 96well sitting-drop vapor diffusion plates (Molecular Dimensions) at 18°C and set up with a mosquito HTS robot (TTP Labtech). Drop ratios of 0.2 μl + 0.2 μl (protein solution + reservoir solution) were used for coarse and fine screening. Hits were obtained under multiple conditions and optimized subsequently.

Data were collected from crystals grown in 0.69 M ammonium sulfate, 200 mM NaCl, 100 mM HEPES pH 7.0. Crystal-containing drops were mixed with 25% glycerol in reservoir solution prior to picking and flash freezing in liquid nitrogen. Diffraction data were collected at the Diamond Light Source (DLS) on beamline I03. Datasets were autoprocessed with XIA2 DIALS, scaled using Aimless (Evans and Murshudov, 2013) and Refmac5 in the CCP4 suite of programs. Structure refinement and manual model building were performed with Refmac5 and COOT (Emsley et al., 2010). Color figures were prepared with PyMol (Schrödinger).

#### Size exclusion chromatography – multi-angle light scattering

Purified recombinant proteins were quantified by NanoDrop using the protein-specific extinction coefficient and diluted to the desired concentration (2 mg/mL unless stated otherwise). SEC-MALS was performed in PBS with 1 mM DTT on a Superdex200 10/300 GL column (GE Healthcare) using an Agilent 1200 Series chromatography system coupled to a DAWN Heleos II multi-angle light scattering detector as well as an Optilab rEX refractive index detector (Wyatt Technology). 100 μl sample was used per run at a flow rate of 0.5 mL/min. BSA was used for calibration. Baseline correction, selection of peaks and calculation of molecular masses was performed with the Astra 6.1 software package.

#### Immunoprecipitation and mass spectrometry

In each experiment, approximately 5ml of *Arabidopsis* seeds was used for the SOK line and for Col-0 as control. Seeds were sterilized as described above and plated on 1/2MS plates covered with nylon mesh. Plates were grown vertically for 5-6 days, in conditions as described above. For affinity purifications with pSOK1::SOK1-YFP, 3 g seedlings were used for each replicate. For pRPS5A::SOK1-YFP, 1.5 g seedling roots was used per replicate, for pRPS5a::SOK2-YFP 1.8g, and for pRPS5a::SOK3-YFP 1.5g. Each experiment contained 3 technical replicates of wt Col-0 as control and 3 replicates of SOK-YFP. IP-MS was performed as described in Wendrich et al. (2017).

Briefly, samples were snap frozen in liquid nitrogen, ground thoroughly in an ice cold mortar, and 9mL EB (0.05M Tris-HCl pH7.5, 0.15M NaCl, 1%NP40, 1 Protease inhibitor tablet (Roche) per 50 ml solution) was added. Samples were sonicated on ice for 3 times 15 s, with at least 15 s pause in between. Proteins were extracted on ice for 30 minutes and subsequently diluted 5x with EB lacking NP40. Diluted samples were centrifuged twice for 15 minutes at 4°C, 18,000 rpm. The supernatant was filtered through a 40μm cell strainer and μMACS Anti-GFP MicroBeads (Miltenyi Biotec) were added. For pSOK1::SOK1-YFP, 100μl of MicroBeads were added, for the pRPS5a::SOK-YFP pull-downs 75 μl was used. The samples were rotated for 2 hours at 4°C to bind protein complexes. μMACS colums (Miltenyi Biotec) were fitted into a μMACS Separator (Miltenyi Biotec) and equilibrated with EB with 0.1% NP40. The protein extracts were run over the columns at 4°C. Next, the columns were washed 4 times with 200 μl EB with 0.1% NP40 and twice with 500 μl 50mM NH_4_HCO_3_ pH8. To elute the samples, 50 μL 50mM NH_4_HCO_3_ pH8, pre-heated at 95°C was applied directly after removing the columns from the magnet.

The eluates were next treated with 10 mM dithiotreitol (DTT) for 2 hours at 60°C, then with 15 mM iodocetamide in for 2 hours at room temperature in the dark. Finally, samples were treated with 4 mM L-cysteine. Samples were incubated overnight at 20°C with 1 μl trypsin sequencing grade (0.5 μg/μl in 1 mM HCl). The next morning, the pH of the samples was set to 3 with trifluoroacetic acid. Samples were cleaned up using homemade columns: 200 μl pipet tips were stuffed with a piece of C18 Empore disk (Thermo) and filled with 200 μl methanol. 4 μl of 50% LiChroprep RP18 (Merck) slurry in methanol was added to the tip. Elution of the columns was performed with a syringe or vacuum manifold until the final elution of the sample, but columns were not allowed to run dry. The column was washed again with methanol. Columns were equilibrated with 100 μl 1 ml/l HCOOH in MiliQ. The samples were centrifuged at maximum speed for 10 minutes to remove beads, and the supernatant was applied to the columns and run through. The columns with bound sample were washed with 100 μl 1 ml/l HCOOH in MiliQ. Finally, samples were manually eluted by applying 50 μl 50% Acetonitrile + 50% 1 ml/l HCOOH to the column and applying pressure with a syringe. The cleaned up samples were collected in low-binding Eppendorf tubes and samples were dried in a SpeedVac at 45°C for at least 2 hours. 50 μl of 1ml/l HCOOH was added to each sample, after which samples were sonicated for 5x 10 seconds in a water bath sonicator with brief vortexing after each sonication step.

Samples were applied to online nanoLC-MS/MS using a 60 min acetonitrile gradient from 8%–50%. Spectra were recorded on a LTQ-XL mass spectrometer (Thermo Scientific).

#### Microscopy of plant specimens

Confocal images of *Arabidopsis* and *Physcomitrella* were taken on a Leica SP5 or SP8 confocal microscope equipped with an Argon laser and DSS561 diode laser (SP5) or white light laser (SP8). YFP was excited at 514 nm, and propidium iodide, mCherry and tdTomato at 561 nm. *Arabidopsis* roots were counterstained with 1 μg/ml propidium iodide (Sigma) for 1-5 minutes. For imaging of *Arabidopsis*, filters were set at ∼520-550nm for YFP, and 600-650nm for tdTomato and propidium iodide. For embryo imaging, ovules were isolated and gently pressed to liberate embryos in PBS buffer. Imaging was performed as described above.

For imaging of *Physcomitrella*, emission light was detected at ∼525-550 nm for mCitrine and at ∼600-650 nm for mCherry. Both naturally occurring *Physcomitrella* gametophore buds and buds induced by 1μM BAP were examined.

Observation of *Marchantia* 35S-mCitrine-MpSOSEKI transgenic lines was performed on gemmae. Citrine was excited with a 514nm wavelength laser and fluorescence was detected at 525-580nm using a Leica SP8 confocal microscope.

The plasmolysis experiment in *Arabidopsis* was performed by dipping roots in a solution of mannitol at a final concentration of 0.4M. Confocal imaging was performed on a Leica SP8 confocal microscope with a hybrid detector in photon counting mode. The following laser settings were used for root observation: YFP (excitation 514nm and emission 525-550nm), tdTomato (excitation 561nm, emission 570-700nm).

#### Quantification of cell division defects in *Arabidopsis*

Selected primary transgenics were transferred to MS plates without herbicide for 3-4 days before imaging. Roots were stained with propidium iodide (Sigma-Aldrich) at a final concentration of 10μg/ml and imaged using a Leica SP8 confocal microscope. Roots were imaged to detect the propidium iodide staining (excitation 561nm, emission 600-700nm) as well as SOK1-YFP expression (excitation 514nm and emission 525-550nm).

#### Immunofluorescence of mammalian cells

COS-7 cells were cultured in 6-well culture dishes and transfected with 0.5μg DNA and 3.5x PEI, grown for 22 hours and fixed on coverslips with 4% formaldehyde in PBS and subsequently permeabilized by 0.5% Triton X-100 in PBS. Cells were blocked in 3% bovine serum albumin in PBS-T for at least 10 minutes, and subsequently incubated with primary antibodies for > 1 hour. Cells were washed in blocking buffer and PBS-T and incubated with secondary antibodies labeled with fluorophores. Coverslips were washed and embedded with VectaShield with DAPI mounting media. Images were acquired with identical settings using a Zeiss 710 Confocal Microscope using ‘best signal’ setting (Smart Setup, ZEN software, Zeiss)

### Quantification and Statistical Analysis

#### Analysis of cell division defects

Roots were scanned for cells with division plane defects in the cell layers expressing the SOSEKI protein. Quantification of cell division defects in *Arabidopsis* was performed by counting the number of T1 generation roots showing aberrant cell division and the total number of roots examined. Root cells from the meristematic region divide orthogonally to the surface of the root. In our growth conditions, we rarely observe a deviation of the plane of division of more than 15° from the primary axis of root growth. Hence, any deviation exceeding 15° was considered an aberrant cell division. The angles were measured using ImageJ. A percentage of defective versus total number of roots was calculated.

#### Analysis of mass-spectrometry data

Data analysis of obtained spectra was done in MaxQuant software package v1.6.8.0 (Tyanova et al., 2016a) as described (Wendrich et al., 2017). Data analysis and visualization was performed in Perseus v1.6.2.3. (Tyanova et al., 2016b), Adobe Illustrator and R.

#### Cell culture experiments

All error bars are represented as mean ± SEM for 6 independent experiments. Statistical significance was calculated in Prism V8.0 (GraphPad) by ANOVA test and denoted as ^∗^ = p < 0.0001 between indicated data points.

### Data and Code Availability

Coordinates for AtSOK4 DIX were deposited with the PDB under 6RSN. Phylogenetic trees were deposited at iTOL (https://itol.embl.de/shared/dolfweijers).
