# Supplementary material for: DIX Domain Polymerization Drives Assembly of Plant Cell Polarity Complexes
Source: Cell. 2020 Feb 6;180(3):427–439.e12. doi: 10.1016/j.cell.2020.01.011 (PMC7042713; doi:10.1016/j.cell.2020.01.011)
Supplement: Document S1. Tables S1–S5 [file mmc1.pdf]

Cell, Volume *180*

Supplemental Information

DIX Domain Polymerization Drives Assembly of Plant Cell Polarity Complexes

Maritza van Dop, Marc Fiedler, Sumanth Mutte, Jeroen de Keijzer, Lisa Olijslager, Catherine Albrecht, Che-Yang Liao, Marcel E. Janson, Mariann Bienz, and Dolf Weijers

Table S1 (Related to Fig. 1): SOSEKI gene distribution in plant genomes

Lineage	Class	Species	Family	GeneID
Angiosperms	Rosids	*Vigna radiata*	Fabaceae	vra_01g10340,vra_02g05090,vra_02g06290,vra_04g03140,vra_08g22610
Angiosperms	Rosids	*Vigna angularis*	Fabaceae	van_0010ss02280.1,van_0022ss04200.1,van_0032ss01130.1,van_0032ss01130.2,van_0032ss01130.3,van_10g03360.1
Angiosperms	Rosids	*Phaseolus vulgaris*	Fabaceae	pvu_002G261900,pvu_005G042900,pvu_005G069000,pvu_005G130600,pvu_011G066900
Angiosperms	Rosids	*Glycine max*	Fabaceae	gma_05G180800,gma_08G138500,gma_08G159000,gma_11G142600,gma_12G065900,gma_12G178300,gma_13G322200,gma_15G267400
Angiosperms	Rosids	*Cajanus cajan*	Fabaceae	cca_14036,cca_21153,cca_21591,cca_25562
Angiosperms	Rosids	*Trifolium pratense*	Fabaceae	tpr_gene1012,tpr_gene16144,tpr_gene21027,tpr_gene2242,tpr_gene28209
Angiosperms	Rosids	*Medicago truncatula*	Fabaceae	mtr_2g062310,mtr_2g084275,mtr_4g057330,mtr_4g063130,mtr_8g091300
Angiosperms	Rosids	*Arachis duranensis*	Fabaceae	adu_Aradu.4PC58,adu_Aradu.AEQ3S,adu_Aradu.CA7FP,adu_Aradu.RWZ7G
Angiosperms	Rosids	*Lotus japonicus*	Fabaceae	lja_0g3v0262389.1lja,lja_3g3v1113340.1lja,lja_3g3v3033280.1lja,lja_3g3v3165170.1lja,lja_4g3v2819920.1lja
Angiosperms	Rosids	*Lupinus angustifolius*	Fabaceae	Lang109325917,Lang109326960,Lang109334206,Lang109347963,Lang109351607,Lang109353497,Lang109353536
Angiosperms	Rosids	*Cicer arietinum*	Fabaceae	car_00770.1,car_02966.1,car_11031.1,car_18456.1,car_24422.1|scaffold01402:27950-31384|plus|
Angiosperms	Rosids	*Prunus mume*	Rosaceae	pmu_000811,pmu_001152,pmu_002067,pmu_016412,pmu_030310
Angiosperms	Rosids	*Prunus persica*	Rosaceae	ppe_2G001400,ppe_6G084700,ppe_6G112400,ppe_6G181200
Angiosperms	Rosids	*Pyrus x bretschneideri*	Rosaceae	pbr_022560.1,pbr_023770.1,pbr_026434.1,pbr_028979.1,pbr_030366.1,pbr_033989.1,pbr_039290.1
Angiosperms	Rosids	*Malus domestica*	Rosaceae	mdo_0000164133,mdo_0000191557,mdo_0000220817,mdo_0000248575,mdo_0000255456,mdo_0000300897,mdo_0000839354
Angiosperms	Rosids	*Rubus occidentalis*	Rosaceae	roc_G02053,roc_G02523,roc_G10699,roc_G21226
Angiosperms	Rosids	*Fragaria vesca*	Rosaceae	fve_13969,fve_16367,fve_24610
Angiosperms	Rosids	*Morus notabilis*	Moraceae	Mnot21394126,Mnot21399641,Mnot21403527,Mnot21408099
Angiosperms	Rosids	*Ziziphus jujuba*	Rhamnaceae	Zjuj107411067,Zjuj107411431,Zjuj107413514,Zjuj107425435
Angiosperms	Rosids	*Humulus lupulus*	Cannabaceae	hlu_G011207,hlu_G020145,hlu_G029486,hlu_G034927
Angiosperms	Rosids	*Jatropha curcas*	Euphorbiaceae	jcu_4S01551.20jcu,jcu_4S01614.10jcu,jcu_4S02133.40jcu,jcu_4S04391.30jcu,jcu_4S06495.20jcu
Angiosperms	Rosids	*Manihot esculenta*	Euphorbiaceae	mes_04G013400,mes_04G030500,mes_10G153300,mes_11G134200,mes_11G152200,mes_12G137400
Angiosperms	Rosids	*Ricinus communis*	Euphorbiaceae	rco_28180.t000021,rco_28653.t000004,rco_29589.t000033,rco_29713.t000010,rco_30226.t000064
Angiosperms	Rosids	*Linum usitatissimum*	Linaceae	lus_10003921,lus_10004943,lus_10005442,lus_10016150,lus_10021417,lus_10037468,lus_10040860
Angiosperms	Rosids	*Populus trichocarpa*	Salicaceae	ptr_001G231700,ptr_001G235700,ptr_004G216200,ptr_005G078300,ptr_009G005800,ptr_009G024900,ptr_009G027500
Angiosperms	Rosids	*Cucumis sativus*	Cucurbitaceae	csa_049870,csa_165470,csa_213490,csa_385720
Angiosperms	Rosids	*Cucumis melo*	Cucurbitaceae	cme_3C020702,cme_3C023839,cme_3C026950
Angiosperms	Rosids	*Citrullus lanatus*	Cucurbitaceae	cla_002398,cla_004028,cla_008280,cla_008702,cla_015291
Angiosperms	Rosids	*Castanea mollissima*	Fagaceae	cmo_s00909_0.44_1,cmo_s02976_0.20_1,cmo_s04551_0.14_1,cmo_s05226_0.14_1,cmo_s06746_0.13_1
Angiosperms	Rosids	*Juglans regia*	Juglandaceae	jre_00004214,jre_00009636,jre_00010373,jre_00011105,jre_00015505
Angiosperms	Rosids	*Betula pendula*	Betulaceae	Bpen1260976288,Bpen1260982609,Bpen1261012322,Bpen1261014530,Bpen1261028988
Angiosperms	Rosids	*Capsella grandiflora*	Brassicaceae	cgr_10637s0002,cgr_1085s0044,cgr_12562s0001,cgr_1317s0037,cgr_1671s0185
Angiosperms	Rosids	*Capsella rubella*	Brassicaceae	cru_10001062,cru_10011656,cru_10017467,cru_10023344,cru_10026455
Angiosperms	Rosids	*Arabidopsis lyrata*	Brassicaceae	AlyrAL1G15460,AlyrAL4G22000,AlyrAL5G24860,AlyrAL6G20610,AlyrAL6G20640,AlyrAL8G36340
Angiosperms	Rosids	*Arabidopsis thaliana*	Brassicaceae	ath_AT2G28150,ath_AT3G46110,ath_AT5G10150,ath_AT5G59790
Angiosperms	Rosids	*Camelina sativa*	Brassicaceae	Csat104700722,Csat104708090,Csat104737118,Csat104739095,Csat104762094,Csat104763394,Csat104769214,Csat104780427,Csat104786906,Csat104790845
Angiosperms	Rosids	*Brassica oleracea*	Brassicaceae	bol_Bo2g009720,bol_Bo2g025400,bol_Bo4g071860,bol_Bo5g006810,bol_Bo8g115040,bol_Bo9g140780
Angiosperms	Rosids	*Brassica rapa*	Brassicaceae	bra_B00345,bra_B01070,bra_G01352,bra_I05446,bra_J00384,bra_J01348
Angiosperms	Rosids	*Brassica napus*	Brassicaceae	bnp_BnaA02g00380D,bnp_BnaA02g06840D,bnp_BnaA02g06850D,bnp_BnaA07g13680D,bnp_BnaA09g50300D,bnp_BnaC02g00480D,bnp_BnaC04g16130D,bnp_BnaC05g03700D
Angiosperms	Rosids	*Raphanus raphanistrum*	Brassicaceae	rra_10931_p1,rra_1916_p3,rra_1937_p5,rra_2002_p1,rra_20660_p2,rra_23335_p1
Angiosperms	Rosids	*Thellungiella halophila*	Brassicaceae	thh_10009387m,thh_10013653m,thh_10013690m,thh_10013702m,thh_10016498m
Angiosperms	Rosids	*Thellungiella salsuginea*	Brassicaceae	tsa_Ts1g04510,tsa_Ts4g11810,tsa_Ts6g23890,tsa_Ts6g36370
Angiosperms	Rosids	*Leavenworthia alabamica*	Brassicaceae	lal_scaffold1335_14lal,lal_scaffold2189_9lal,lal_scaffold3078_5lal,lal_scaffold432_5lal,lal_scaffold450_51lal,lal_scaffold493_20lal,lal_scaffold620_45lal
Angiosperms	Rosids	*Aethionema arabicum*	Brassicaceae	aar_AA16G00068,aar_AA32G00886,aar_AA44G00278,aar_AA4G00026,aar_AA97G00030
Angiosperms	Rosids	*Schrenkiella parvula*	Brassicaceae	spa_c0001_00391,spa_c0002_00815,spa_c0002_01885,spa_c0004_01864,spa_c0117_00010
Angiosperms	Rosids	*Boechera stricta*	Brassicaceae	Bostr.13175s0086,Bostr.18473s0332,Bostr.25219s0413,Bostr.26833s0258,Bostr.27991s0113
Angiosperms	Rosids	*Arabis alpina*	Brassicaceae	Alp318770,Alp356480,Alp504030,Alp79410,Alp821710
Angiosperms	Rosids	*Sisymbrium irio*	Brassicaceae	sir_scaffold1277_13sir,sir_scaffold1460_2sir,sir_scaffold2746_51sir,sir_scaffold418_8sir
Angiosperms	Rosids	*Cleome gynandra*	Cleomaceae	cgy_12555,cgy_12664,cgy_13985,cgy_14990,cgy_15372
Angiosperms	Rosids	*Tarenaya hassleriana*	Cleomaceae	tha_Th2v07633,tha_Th2v13633,tha_Th2v15660,tha_Th2v15972,tha_Th2v18805,tha_Th2v21279

Angiosperms	Rosids	*Carica papaya*	Caricaceae	cpa_1.354cpa,cpa_190.33cpa,cpa_476.3cpa
Angiosperms	Rosids	*Gossypium raimondii*	Malvaceae	gra_001G108000,gra_003G136300,gra_003G146100,gra_004G232000,gra_007G241300,gra_009G101600
Angiosperms	Rosids	*Theobroma cacao*	Malvaceae	tca_001159,tca_036521,tca_041902,tca_042162,tca_042839
Angiosperms	Rosids	*Eucalyptus grandis*	Myrtaceae	egr_B02400,egr_G03434,egr_I00418,egr_J00387
Angiosperms	Rosids	*Citrus sinensis*	Rutaceae	csi_1g009456,csi_1g015170,csi_1g015443,csi_1g048680
Angiosperms	Rosids	*Vitis vinifera*	Vitaceae	vvi_01005997001,vvi_01006373001,vvi_01016603001,vvi_01025033001,vvi_01031469001
Angiosperms	Super-Asterids	*Solanum pennellii*	Solanaceae	spe_01g033180.1,spe_04g024600.1,spe_06g032080.1,spe_11g026130.1,spe_11g026130.2,spe_11g030760.1
Angiosperms	Super-Asterids	*Solanum lycopersicum*	Solanaceae	sly_01g081630.2sly,sly_04g010330.2sly,sly_06g030470.2sly,sly_06g075690.2sly,sly_11g066380.1sly,sly_11g073110.1sly
Angiosperms	Super-Asterids	*Solanum tuberosum*	Solanaceae	stu_00000416,stu_00002267,stu_00015560,stu_00020631,stu_00030309,stu_00031117
Angiosperms	Super-Asterids	*Solanum melongena*	Solanaceae	sme_00019.1_g00004.1,sme_00133.1_g00016.1,sme_00896.1_g00009.1,sme_03876.1_g00006.1,sme_06578.1_g00002.1
Angiosperms	Super-Asterids	*Capsicum annuum*	Solanaceae	can_Capana01g003241,can_Capana06g000459,can_Capana11g000172,can_Capana11g000756,can_Capana12g001628
Angiosperms	Super-Asterids	*Nicotiana benthamiana*	Solanaceae	nbe_NbC25559599g0003,nbe_NbS00005205g0002,nbe_NbS00012706g0013,nbe_NbS00018660g0016,nbe_NbS00039215g0008,nbe_NbS00050054g0014
Angiosperms	Super-Asterids	*Nicotiana tomentosiformis*	Solanaceae	Ntom104098369,Ntom104102300,Ntom104106065,Ntom104111073,Ntom104111968,Ntom104112606
Angiosperms	Super-Asterids	*Nicotiana attenuata*	Solanaceae	Nattg00112,Nattg02993,Nattg14428,Nattg21505,Nattg35714,Nattg37750
Angiosperms	Super-Asterids	*Nicotiana sylvestris*	Solanaceae	Nsyl104211961,Nsyl104212342,Nsyl104218199,Nsyl104231073,Nsyl104239700,Nsyl104243301
Angiosperms	Super-Asterids	*Petunia axillaris*	Solanaceae	pax_00365g00021,pax_00402g00927,pax_00411g00116,pax_00422g00079,pax_00983g00415,pax_00986g00225
Angiosperms	Super-Asterids	*Ipomoea nil*	Convolvulaceae	Inil109148068,Inil109172632,Inil109178484,Inil109185924,Inil109193029
Angiosperms	Super-Asterids	*Utricularia gibba*	Lentibulariaceae	ugi_Scf00042.g4844ugi,ugi_Scf00082.g7495ugi,ugi_Scf00117.g9308ugi,ugi_Scf00161.g11077ugi,ugi_Scf00197.g12222ugi,ugi_Scf01019.g23292ugi
Angiosperms	Super-Asterids	*Sesamum indicum*	Pedaliaceae	sin_1004336,sin_1005176,sin_1008257,sin_1010486,sin_1024084,sin_1024192
Angiosperms	Super-Asterids	*Mimulus guttatus*	Phrymaceae	mgu_L00105,mgu_N01652,mgu_N02545,mgu_N02662
Angiosperms	Super-Asterids	*Coffea canephora*	Rubiaceae	coc_01_g10330,coc_01_g12290,coc_02_g08340,coc_02_g26760
Angiosperms	Super-Asterids	*Lactuca sativa*	Asteraceae	Lsat1_64580,Lsat1_87440,Lsat5_167421,Lsat5_42720,Lsat6_43200,Lsat9_22261
Angiosperms	Super-Asterids	*Helianthus annuus*	Asteraceae	hel_Ha1_00043918,hel_Ha12_00033351,hel_Ha13_00014838,hel_Ha15_00040114,hel_Ha2_00028128,hel_Ha4_00025522,hel_Ha8_00042287
Angiosperms	Super-Asterids	*Daucus carota*	Apiaceae	dca_006003,dca_011505,dca_015507,dca_018884,dca_024996,dca_029581
Angiosperms	Super-Asterids	*Actinidia chinensis*	Actinidiaceae	ach_Achn067311,ach_Achn107861,ach_Achn171521,ach_Achn258271,ach_Achn295901,ach_Achn378401
Angiosperms	Super-Asterids	*Chenopodium quinoa*	Amaranthaceae	CquiAUR62001837,CquiAUR62002030,CquiAUR62003768,CquiAUR62009625,CquiAUR62014960,CquiAUR62029174
Angiosperms	Super-Asterids	*Spinacia oleracea*	Amaranthaceae	sol_142250_rhzn.t1,sol_180050_gfew.t1
Angiosperms	Super-Asterids	*Beta vulgaris*	Amaranthaceae	bvu_Bv6_135470_ofdn,bvu_Bv7_174520_gnot,bvu_Bv9_205850_sofh
Angiosperms	Super-Asterids	*Amaranthus hypochondriacus*	Amaranthaceae	AhypAH000219,AhypAH005948,AhypAH013139,AhypAH022517
Angiosperms	Eudicots	*Nelumbo nucifera*	Nelumbonaceae	nnu_03010,nnu_12336,nnu_16954,nnu_20223,nnu_22447,nnu_23257,nnu_23590
Angiosperms	Monocots	*Triticum urartu*	Poaceae	tur_TRIUR3_07098,tur_TRIUR3_24591,tur_TRIUR3_26826,tur_TRIUR3_32429
Angiosperms	Monocots	*Triticum aestivum*	Poaceae	tae_Traes_2BS_C2A6130B11,tae_TRAES3BF060000010CFD,tae_TRAES3BF065400020CFD,tae_TRAES3BF127400020CFD,tae_Traes_4AL_9E250501B,tae_Traes_4AS_048C1EC18,tae_Traes_4DL_62077A769,tae_Traes_4DS_4326E7827
Angiosperms	Monocots	*Aegilops tauschii*	Poaceae	ata_EMT02390,ata_EMT08241,ata_EMT10074,ata_EMT27042,ath_AT1G05577
Angiosperms	Monocots	*Hordeum vulgare*	Poaceae	HORVU3Hr1G076430,HORVU3Hr1G086700,HORVU3Hr1G116730,HORVU4Hr1G005250,HORVU7Hr1G045060
Angiosperms	Monocots	*Brachypodium distachyon*	Poaceae	bdi_Bradi1g12010,bdi_Bradi1g71710,bdi_Bradi2g51680,bdi_Bradi2g54510,bdi_Bradi2g62490,bdi_Bradi3g43040
Angiosperms	Monocots	*Oryza glaberrima*	Poaceae	ogl_01G0270100,ogl_01G0304800,ogl_03G0064900,ogl_03G0277600,ogl_04G0281500,ogl_08G0191100
Angiosperms	Monocots	*Oryza sativa*	Poaceae	osa_LOC_Os01g57020osa,osa_LOC_Os01g62200osa,osa_LOC_Os03g09200osa,osa_LOC_Os03g48600osa,osa_LOC_Os08g44760osa
Angiosperms	Monocots	*Oryza rufipogon*	Poaceae	oru_ORUFI01G36150.1oru,oru_ORUFI03G06610.1oru,oru_ORUFI03G31600.1oru,oru_ORUFI08G26200.1oru,oru_ORUFI09G15590.1oru
Angiosperms	Monocots	*leersia perrieri*	Poaceae	lpe_01G28490.1lpe,lpe_01G31510.1lpe,lpe_03G06010.1lpe,lpe_03G25060.1lpe,lpe_08G20420.1lpe
Angiosperms	Monocots	*Phyllostachys heterocycla*	Poaceae	phe_PH01000129G0220,phe_PH01000374G0210,phe_PH01000788G0630,phe_PH01001206G0640,phe_PH01001642G0360,phe_PH01002026G0070
Angiosperms	Monocots	*Zea mays*	Poaceae	zma_GRMZM2G070442,zma_GRMZM2G086123,zma_GRMZM2G112836,zma_GRMZM2G136710,zma_GRMZM2G177052,zma_GRMZM5G871592
Angiosperms	Monocots	*Zea mays -V4*	Poaceae	Zmay100272305_1,Zmay100272305_2,Zmay100272305_3,Zmay100276675_1,Zmay100276675_2,Zmay103636515_1,Zmay103636515_2,Zmay103636515_3,Zmay103643835,Zmay103651032
Angiosperms	Monocots	*Sorghum bicolor*	Poaceae	sbi_001G130700,sbi_001G473500,sbi_003G315600,sbi_003G349800,sbi_007G166800
Angiosperms	Monocots	*Setaria italica*	Poaceae	sit_5G339100,sit_5G375200,sit_6G251500,sit_9G132200,sit_9G509000
Angiosperms	Monocots	*Oropetium thomaeum*	Poaceae	oth_00349,oth_10088,oth_12702,oth_14464,oth_18829
Angiosperms	Monocots	*Ananas comosus*	Bromeliaceae	aco_004096,aco_009327,aco_010974,aco_020392,aco_023608
Angiosperms	Monocots	*Elaeis guineensis*	Arecaceae	egu_sc00069_439,egu_sc00099_250,egu_sc00134_228,egu_sc00140_191
Angiosperms	Monocots	*Phoenix dactylifera*	Arecaceae	Pdac103696515,Pdac103705503,Pdac103705998,Pdac103708106,Pdac103717242,Pdac103719979
Angiosperms	Monocots	*Musa acuminata*	Musaceae	mac_01_02060.1mac,mac_02_23990.1mac,mac_05_14790.1mac,mac_05_30710.1mac,mac_05_30900.1mac,mac_09_29020.1mac,mac_10_19000.1mac,mac_11_02160.1mac,mac_11_22820.1mac

Angiosperms	Monocots	*Dendrobium catenatum*	Orchidaceae	Dcat110091826,Dcat110099896,Dcat110099918,Dcat110103181,Dcat110105435,Dcat110111944,Dcat110115073
Angiosperms	Monocots	*Phalaenopsis equestris*	Orchidaceae	peq_10763,peq_14560,peq_17260,peq_19806,peq_21055,peq_40778
Angiosperms	Monocots	*Asparagus officinalis*	Asparagaceae	Aoff109822809,Aoff109832142,Aoff109834999,Aoff109836511,Aoff109843594
Angiosperms	Monocots	*Xerophyta viscosa*	Velloziaceae	Xvis03_200935,Xvis03_201836,Xvis03_202066,Xvis03_213866,Xvis03_214169,Xvis03_218847
Angiosperms	Monocots	*Spirodela polyrhiza*	Araceae	spo_17G0045300,spo_1G0018300,spo_23G0024700,spo_4G0012900
Angiosperms	Monocots	*Lemna minor*	Lemnaceae	lmi_001103,lmi_017315
Angiosperms	Monocots	*Zostera marina*	Zosteraceae	zom_15g01360,zom_241g00190,zom_2g02660,zom_303g00130,zom_43g00720
Angiosperms		*Amborella trichopoda*	Amborellaceae	atr_scaffold00068.91atr,atr_scaffold00089.37atr,atr_scaffold00178.7atr
Gymnosperms		*Gnetum montanum*	Gnetaceae	GMO00027921
Gymnosperms		*Picea abies*	Pinaceae	PAB00014665, PAB00038888, PAB00040409
Gymnosperms		*Picea glauca*	Pinaceae	PGL00019156, PGL00019193
Gymnosperms		*Pseudotsuga menziesii*	Pinaceae	PME00003895, PME00004197, PME00113392
Gymnosperms		*Pinus pinaster*	Pinaceae	PPI00000548, PPI00003747, PPI00007365, PPI00014188, PPI00031688
Gymnosperms		*Pinus sylvestris*	Pinaceae	PSY00017750, PSY00018635, PSY00018636
Gymnosperms		*Populus trichocarpa*	Salicaceae	PT01G23170, PT01G23230, PT01G23570, PT04G21620, PT05G07830, PT08G05100, PT09G00580, PT09G02490, PT09G02750, PT10G20960
Gymnosperms		*Pinus taeda*	Pinaceae	PTA00006616, PTA00017601, PTA00021624, PTA00027577, PTA00039081, PTA00043528, PTA00058226, PTA00063752, PTA00066655, PTA00081843
Gymnosperms		*Taxus baccata*	Taxaceae	TBA00021570, TBA00022943, TBA00025016
Lycophyte		*Selaginella mollendorfii*		Sm412562, Sm439304, Sm409423, Sm449230

Table S2 (Related to Fig. 4): Data collection and refinement statistics

**Data Collection**	**AtSOK1 DIX (6RSN)**
Space Group	P 61
Cell dimensions	
a, b, c (Å)	47.66, 47.66, 67.97
α, β, γ (º)	90.0, 90.0, 120.0
Resolution (Å)	35.28 - 1.70
*Rmerge (%)	7.5 (65.9)
Mean I/σ(I)	33.91
Completeness (%)	99.5
Multiplicity	20.0
Complexes in A.U.	1
**Refinement**	
Resolution (Å)	35.28 - 1.70
No. Reflections	192374
R_{work} / R_{free} (%)	28.5 / 21.4
No. Atoms	808
Wilson B-factor (Å^2)	25.5
Anisotropy	0.154
Average B, all atoms (Å^2)	38.0
B-factors	
Protein	27.6
Ligands	72.8
Water	40.9
R.m.s. deviations	
Bond length (Å)	0.014
Bond angle (º)	2.22

* Highest resolution shell (in Å) shown in parenthesis.

Table S3 (Related to Fig. 7): DIX domain proteins used in this study

GeneName	Species	UniprotID/GeneID
DVL1	*M. musculus*	P51141
DVL2	*M. musculus*	Q60838
DVL3	*M. musculus*	Q61062
DVL1	*H. sapiens*	O14640
DVL2	*H. sapiens*	O14641
DVL3	*H. sapiens*	Q92997
DVL2	*X. laevis*	P51142
DVL3	*X. laevis*	Q6DKE2
DSH	*D. melanogaster*	P51140
DSH-MIG5	*C. elegans*	Q22227
DSH	*D. rerio*	F1QM97
AXIN1	*M. musculus*	O35625
AXIN2	*M. musculus*	O88566
AXIN1	*H. sapiens*	O15169
AXIN2	*H. sapiens*	Q9Y2T1
AXIN1	*D. rerio*	P57094
AXIN1	*G. domesticus*	O42400
AXIN1	*X. laevis*	Q9YGY0
AXN	*D. melanogaster*	Q9V407
AXN-PRY1	*C. elegans*	O62090
DIXC1	*M. musculus*	Q80Y83
DIXC1	*H. sapiens*	Q155Q3
DIX1A	*D. rerio*	Q804T6
MpSOK	*M. polymorpha*	Mapoly0032s0007
PpSOK1	*P. patens*	Pp3c14_23220V3.1
PpSOK2	*P. patens*	Pps3c2_20380V3.1
PpSOK3	*P. patens*	Pp3c17_23930V3.1
PpSOK4	*P. patens*	Pp3c18_11140V3.1
PpSOK5	*P. patens*	Pp3c1_41700V3.1
PpSOK6	*P. patens*	Pp3c19_18440V3.1
PpSOK7	*P. patens*	Pp3c22_12010V3.1
PpSOK8	*P. patens*	Pp3c7_12520V3.1
PpSOK9	*P. patens*	Pp3c1_15970V3.1
AtSOK1	*A. thaliana*	AT1G05577
AtSOK2	*A. thaliana*	AT5G10150
AtSOK3	*A. thaliana*	AT2G28150
AtSOK4	*A. thaliana*	AT3G46110
AtSOK5	*A. thaliana*	AT5G59790
	P. falciparum	XP_008892787
	P. tetraurelia	A0DFT1
	C. parvum	XP_626730
	P. falciparum	XP_001348377
	B. hominis	XP_012898418
	E. siliculosus	CBN75002

Table S4 (Related to STAR Methods): Oligonucleotides used in this study

**RPS5A-SOK1(/5WT/mutant)-YFP**	
SOK1 CDS LIC F	TAGTTGGAATAGGTTCATGGAAAGTAATGGTGGAGG
YFP LIC R	AGTATGGAGTTGGGTTCTTACTTGTACAGCTCGTCC
SOK1 C233A F	CGGAACTTGATGAAGGCTGGTGGTTTGGACACA
SOK1 C233A R	TGTGTCCAAACCACCAGCCTTCATCAAGTTCCG
SOK1 C233A/G234A F	CGGAACTTGATGAAGGCTGCTGGTTTGGACACAAAC
SOK1 C233A/G234A R	GTTTGTGTCCAAACCAGCAGCCTTCATCAAGTTCCG
SOK1 C307A/C310A F	TCCATGGCTCCATTAGCCTCGCAAGCTGGGAAGTTGTTCAAA
SOK1 C307A/C310A R	TTTGAACAACTTCCCAGCTTGCGAGGCTAATGGAGCCATGGA
SOK1 D78R F	GAATGGTTATGTATGGCAAAGATTATTGGACGATGATCTTAT
SOK1 D78R R	ATAAGATCATCGTCCAATAATCTTTGCCATACATAACCATTC
SOK1 D78A F	CAAGCCTTATTGGACGATGATC
SOK1 D78A R	CAATAAGGCTTGCCATACATAACC
SOK1 H29D F	CGGGTCACGTTGACCATCCTGATCTCTTGCGTGTTCATCATCT
SOK1 H29D R	AGATGATGAACACGCAAGAGATCAGGATGGTCAACGTGACCCG
Dvl DIX LIC F	TAGTTGGAATAGGTTCATGGCGGGTAGCAGCACTGG
Dvl DIX SOK1 FW	CTCCAGTCCATGAGCCTCGGGCTTGGGTGACAAGAAACGG
Dvl DIX SOK1 RV	CCGTTTCTTGTCACCCAAGCCCGAGGCTCATGGACTGGAG
SOK5 CDS LIC F	TAGTTGGAATAGGTTCATGAGTTCAAGAGTGTTCAGAGC
SOK5 C303A F	GTTAATGCAGCTGATATCGGCTGGTACGATGTCGTTTAAAG
SOK5 C303A R	CTTTAAACGACATCGTACCAGCCGATATCAGCTGCATTAAC
SOK5 E356Q/E359Q F	GGAAGGGTACAATTGCAAGATAAGCAGTATTTTAGCGGCAG
SOK5 E356Q/E359Q R	CTGCCGCTAAAATACTGCTTATCTTGCAATTGTACCCTTCC
**ANGUSTIFOLIA-tdTomato**	
Angustifolia F	TAGTTGGAATGGGTTCTACAACCAAATCGTACCACA
Angustifolia R	TTATGGAGTTGGGTTCCATCGATCCAACGTGTGATAC
**PpSOK-mCitrine fusions (LF, left flank; RF, right flank; Restriction sites underlined)**	
PpSOK1-LF-F-KpnI	GGGGTACCCGCAGAAGCAGAAGTTGACACC
PpSOK1-LF-R-XhoI	CCGCTCGAGGCATCGGACCACATGCGG
PpSOK1-RF-F-SmaI	GGGATTCGTTATTTGTGCGGCTGG
PpSOK1-RF-R-NotI	AGAAGCGGCCGCTTGGTTCTGCTCCCTCCATCG
PpSOK2-LF-F-ApaI	ACGCGGGCCCACCAGAACAAGTCACAGGGAGG
PpSOK2-LF-R-ClaI	CCATCGATTGCAGTATCAAATCCTTTGGAGATTG
PpSOK2-RF-F-SmaI	GGGATGCTCGTAAGTGCCTCATTCAAG
PpSOK2-RF-R-NotI	AGAAGCGGCCGCATGCAACATGGGCGGATACAG
PpSOK2-LF-F-ApaI	ACGCGGGCCCATTGCATTCCTCCTCCCTTGTG
PpSOK3-LF-R-HindIII	CCCAAGCTTCGAAGCTCGAGATCGGACGACG
PpSOK3-RF-F-SmaI	GGGCTTTTTCCTGACTGCCGATTCATGG

PpSOK3-RF-R-NotI	AGAAGCGGCCGCTACATTCTTTCTGCCCACGTCC
PpSOK4-LF-F-KpnI	GGGGTACCCTGCACACAATCTCCCACTCTC
PpSOK4-LF-R-XhoI	CCGCTCGAGTTTTTTGATACTCTGATCCTGTTTTAAGG
PpSOK4-RF-F-SmaI	GGGCCTAGGTATTTGAATTCTGAAGTATCC
PpSOK4-RF-R-NotI	AGAAGCGGCCGCGGTGTCGATGCCTCTTTCGATTG
**PpSOK genotyping primers (before: before insertion site, after: after insertion site)**	
Physco geno mCIT RV	GCCGGACACGCTGAACTTGTG
Physco T35S F	TGCTAAGGCAGGGTTGGTTACG
PpSOK1 FW before	GTCAGTGAACAGGCTTCAC
PpSOK1 RV after	GGTTCGAAATCACTTCCGG
PpSOK2 FW before	CAGACGATCAATAAGGAGGGCACC
PpSOK2 RV after	GTCGAGTAGGGTGCGGGGAG
PpSOK3 FW before	GCCGACATCCCGAAATCTGCC
PpSOK3 RV after	GTTGGGCCTTGTATTCAATAAAG
PpSOK4 FW before	CTGCTTGATAGTCTTTGATAATG
PpSOK4 RV after	CAAGCCCCGCCAAGGTGATGCA
35S-mCitrine-MpSOSEKI	
CA74-MpSOSEKI fw cacc:	caccATGGTTCTGGTAGGTCAAGGTATG
CA75-MpSOSEKI rv stop	TTATTGCAGTCGAGTCCGTATG

Table S5: DIX domain sequences (Related to STAR Methods)

Constructs derived from gene synthesis (gBlocks)<br><br>X: amino acid remaining from TEV protease cut<br>GAMAGSSTGGGG and NPQPEMAPPVHEPR: non-native flanking sequences
Cryptosporidium parvum<br>GAGGGTCTTATCACTGTGTACTACTTCATTCCATCGGATGGCGACAAGGAAAACAACC<br>CCAACACTTTCAAGGTTAAGGGAACCCACTCTACTCTGACGACAAAACAAATTAAGGA<br>TAGCTTCCCGCTGCCAGGAATTTACTATTTCGCTTCAAGGTTCGCGTCGGCAACACGT<br>ATGATGGATGGACCCCTTAAATGACGATGACATTGTTCCATTATATGACGATGCAATT<br>ATCGCCAAAGTTCTTCGTATC<br><br>**Protein:**<br>SNEGLITVYYFIPSDGDKENNPNTFKVKGTHSTLTTKQIKDSFPLPGIYYFRFKVRVGNTYA<br>WMDPLNDDDIVPLYDDAIIAKVLRIN
Paramecium tetraurelia<br>AAGCAATTTACGCTGATTTATTACGTTGTCCCTCAAGATAACGATGATGTCGAAATTCC<br>CAACGCATTTGGTATCGGTAAACAGGTAGACCAAATCACACTGAAGGACATCAAAACT<br>TCTTTTCCGCTTCAGGGAGAATATATTTTCCGCTTCCGCTACAAGACTTCGCATAATAC<br>TGTCTGGTTGGACTTGCCAACTGACACCGCACAGATTCCATTATTTAATTCGCGTATTC<br>TTATTAAGGCGACACGTATT<br><br>**Protein:**<br>MKQFTLIYYVVPQDNDDVEIPNAFGIGKQVDQITLKDIKTSFPLQGEYIFRFRYKTSHNTVW<br>LDLPTDTAQIPLFNSRILIKATRIS
Ectocarpus siliculosus<br>ATCCGCTATTTATTCCAGCGGATGGCGACGACCCGTCGCACCCCAACGTTTTTCAGTT<br>GCCCTCTGGCCTGGTCGGAAGCGGGAGTGTACGTTGTGGCGACGTAGAACGTCACTTT<br>CCATTACCGGGTCGTTACCACTTTCGCTTCAAGAAGAAGTTCCGCGATGCTTTTGTGTG<br>GGTAGACATTGCTGATCCCGGCGCGGCAGTCCCAAGTTGTGATGGAGTGTTCACGGCC<br>AAAATC<br><br>**Protein:**<br>MIRYFIPADGDDPSHPNVFQLPSGLVGSGSVRCGDVERHFPLPGRYHFRFKKKFRDAFVW<br>VDIADPGAAVPSCDGVFTAKITRLSD
Plasmodium falciparum<br>ACCGTGGTGTTTTATCATATTATTAACGATAAAGAAGATAAAAACAGCCAGAACGTGT<br>TTTATATTCTGAAACCGATTGGCAGCATTACCCTGAAAGATATTAAACATGAATTTCCG<br>CTGATGGGCACCTATCATTTTCGCTTTAAAATTCTGCATAACAACATTCCGGCGTGGGT<br>GGATGTGACCGATGAAAGCAGCCCGGTGCCGAGCCTGAACAGCTGCATTTATGCGAA<br>AGTGCTGCGCCTG<br><br>**Protein:**<br>GAMAGSSTGGGGKPTVVFYHIINDKEDKNSQNVFYILKPIGSITLKDIKHEFPLMGTYHFRF<br>KILHNNIPAWVDVTDEGSPVPSLNSCIYAKVLRLSNPQPEMAPPVHEPR

Tetrahymena thermophila
AAGGTTATTTCCAACGTGTTCTACCATATTCCTCAGGACAAGGACGACCCCGACATTCC
TAATGTCTTCTGTGTCCCCAAGAGCCAGGAAGAAATTCGTCTTGCAGACATCAAGTCC
TACTTTCCTCTTAATGGTCAATATATCTTCCGCTTTAAGTATCGTTATAACAACCAAGTT
GTCTGGATGGACATCCCGGAAAATTCTAAGAAACTGCCTATTTATGAGGGCCGTATTT
TTCTGAAGGCGACGCGCGTTCAG

Protein:
GAMAGSSTGGGGKVISNVFYHIPQDKDDPDIPNVFCVPKSQEEIRLADIKSYFPLNGQYIFR
FKYRYNNQVVWMD IPENSKKLPIYEGRIFLKATRVQNPQPEMAPPVHEPR

Blastocystis hominis
AAAAAGACTAGTGTGTTGTATTATATCCCCGAGGACGGGGATGAAGCCGACCATCCGA
ATGTAATGACACTGGATAAAAATGCCTCTCAGGTGACGTTGAAGGATATCCGCGAAGC
GTTTCCGATCCCTGGGACGTATTATTTTCGCTTCAAACGTACCTTCAAAAATAGCTGGA
TTTGGTTTGACGTTACTGAGGATTCGGAAGTAGTTCCGAAATTTGACGGTCTGATCTTT
GTAAAAGCGACGCGC

Protein:
GAMAGSSTGGGGSKKTSVLYYIPEDGDEADHPNVMTLDKNASQVTLKDIREAFPIPGTYY
FRFKRTFKNSWIWFDVTEDSEVVPKFDGLIFVKATRLNPQPEMAPPVHEPR

Marchantia polymorpha
GTGGTGTACTACCTTTCACGCGGAGGGCAATTGCAACAGCCCCATCTGATTGATGTCC
CCGTGAGCACCCACTCTAACGGATTGTACTTACGCGACGTAAAACGTCGTCTTACCAG
CATTCGCGGAAAAGGAATGGGGGATTCATTTTCATGGAGTTGCAAGCGCAACTATAAA
AACAATTTCATCTGGCAGGACTTGGCAGACGATGATAAAATTCTTCCTCTTAGTGACG
GTGAACTTGTGCTGAAAGGTTCGGAGTTGTAC

Protein:
GTKVQVVYYLSRGGQLQQPHLIDVPVSTHSNGLYLRDVKRRLTSIRGKGMGDSFSWSCKR
NYKNNFIWQDLADDDKILPLSDGELVLKGSELYTGFQEKAE

Physcomitrella patens SOK2
GTGATTTATCTTCTTTCAAAAGGAGCAGAGCAAGATGATCATCCGCACATGATTCAAG
TGCAGTACCCAAGTCACCAGCACGCACCGACGCTGCGCGATGTCAAGTTCCGTTTAAC
GGCCCTTCGCGGGCGTGGAATGCCAGACTCCTATTCGTGGTCCTATAAGCGTTCCTACA
AGGGTACATTCATTTGGTGTGACGTATTCGATGGAGACGACATCCTGCCG
CTGAGTGAA

Protein:
SESYHKIEVIYLLSKGAEQDDHPHMIQVQYPSHQHAPTLRDVKFRLTALRGRGMPDSYSW
SYKRSYKGTFIWCDVFDGDDILPLSESGEYVLKALEVMD

Constructs derived by PCR from cDNA's

Arabidopsis thaliana SOK1
MESNGGGGEVRRVNLVYFLSRSGHVDHPHLLRVHHLSRNGVFLRDVKKWLADARGDAM
PDAFSWSCKRRYKNGYVWQDLLDDDLITPISDNEYVLKGSEILLSSPKEDYPNVEKK

Arabidopsis thaliana SOK3<br>GKYHQKIKKVQIVYYLSKNRQLEHPHFMEVLISSPNGLYLRDVIERLNVLRGRGMASMYS<br>WSSKRSYRNGFVWHDLSEDDLILPANGNEYVLKGSELFDESNSD
Arabidopsis thaliana SOK4<br>SRERIVPVVYYLSRNGRLDHPHFIEVPLSSHNGLYLKDVINRLNDLRGNGMACLYSWSSKR<br>TYKNGFVWYDLSDEDFIFPVHGQEYVLKGSQILD
